# The sclerostin-neutralizing antibody AbD09097 recognizes an epitope adjacent to sclerostin's binding site for the Wnt co-receptor LRP6

**DOI:** 10.1098/rsob.160120

**Published:** 2016-08-24

**Authors:** V. Boschert, C. Frisch, J. W. Back, K. van Pee, S. E. Weidauer, E.-M. Muth, P. Schmieder, M. Beerbaum, A. Knappik, P. Timmerman, T. D. Mueller

**Affiliations:** 1 Department of Molecular Plant Physiology and Biophysics, Julius-von-Sachs Institute of the University Wuerzburg, Julius-von-Sachs-Platz 2, 97082 Wuerzburg, Germany; 2 Bio-Rad AbD Serotec, Zeppelinstr. 4, 82178 Puchheim, Germany; 3 Pepscan Therapeutics, Zuidersluisweg 2, 8203RC, Lelystad, The Netherlands; 4 Leibniz Institute for Molecular Pharmacology, Robert-Roessle Str. 10, 13125 Berlin, Germany

**Keywords:** sclerostin, neutralizing antibody, osteoporosis, phage display, Wnt signalling

## Abstract

The glycoprotein sclerostin has been identified as a negative regulator of bone growth. It exerts its function by interacting with the Wnt co-receptor LRP5/6, blocks the binding of Wnt factors and thereby inhibits Wnt signalling. Neutralizing anti-sclerostin antibodies are able to restore Wnt activity and enhance bone growth thereby presenting a new osteoanabolic therapy approach for diseases such as osteoporosis. We have generated various Fab antibodies against human and murine sclerostin using a phage display set-up. Biochemical analyses have identified one Fab developed against murine sclerostin, AbD09097 that efficiently neutralizes sclerostin's Wnt inhibitory activity. *In vitro* interaction analysis using sclerostin variants revealed that this neutralizing Fab binds to sclerostin's flexible second loop, which has been shown to harbour the LRP5/6 binding motif. Affinity maturation was then applied to AbD09097, providing a set of improved neutralizing Fab antibodies which particularly bind human sclerostin with enhanced affinity. Determining the crystal structure of AbD09097 provides first insights into how this antibody might recognize and neutralize sclerostin. Together with the structure–function relationship derived from affinity maturation these new data will foster the rational design of new and highly efficient anti-sclerostin antibodies for the therapy of bone loss diseases such as osteoporosis.

## 1. Introduction

Bone is not a dead tissue, but undergoes a permanent adaptation throughout life. In fact, bone modelling occurs continuously to react to differences in mechanical load as well as physiological changes and is not just limited to remodelling after fracture repair. In adulthood, bone formation and bone degradation are usually balanced. At the cellular level this is achieved by fine-tuning the activity of two ‘opposing’ cell types, the bone-forming osteoblasts and the bone-resorbing osteoclasts. Osteocytes, a third class of cells, act as master regulators controlling the activity of osteoblasts and osteoclasts through different hormones and signalling cascades. Disturbing this equilibrium will inevitably lead to pathological conditions. One such example is osteoporosis, which manifests itself by a low bone mineral density leading to a high fracture probability. The disease particularly affects ageing women past menopause probably due to the change in estrogen levels, but also men can suffer from idiopathic osteoporosis. Until recently, the majority of therapeutic approaches against osteoporosis acted to prevent further bone loss usually by targeting the bone-resorbing osteoclasts [1]. Therefore, new treatments that stimulate bone formation and restore initial bone strength are actively pursued in pharmaceutical research and development.

Osteocytes express a protein called sclerostin [2,3], whose name derives from the disease sclerosteosis [4], a rare severe and progressive craniotubular hyperostosis with an autosomal recessive inheritance. Sclerosteosis patients lack sclerostin due to homozygous mutations in the sclerostin-encoding gene *SOST* [2,5], but heterozygous carriers have an increased bone mineral density suggesting a gene dosage effect for sclerostin [6]. In the related van Buchem disease, an enhancer element for *SOST* expression is silenced [7,8]. The most prominent phenotype of both diseases is a progressive bone overgrowth leading to high bone mass, fracture resistance, gigantism and distortion of the facial features (for reviews, see [9,10]), indicating that sclerostin is a negative regulator of bone formation. It was shown that sclerostin inhibits Wnt signalling [11,12], an important pathway for bone formation and bone remodelling (for reviews, see [13,14]). Mutations in the genes of Wnt proteins like Wnt1, Wnt3a, Wnt5a, Wnt10b and Wnt16 in humans or mice either result in low bone mass or affect bone mineral density denoting that these Wnt factors are required for proper bone formation [15–20]. In canonical Wnt signalling, Wnt proteins bind to a receptor of the Frizzled family and to the coreceptor LRP5/6 leading to stabilization of the intracellular protein β-catenin. The latter then translocates to the nucleus where it acts as transcriptional co-activator for Wnt-responsive genes (for reviews, see [21,22]). Sclerostin abrogates this signalling by its ability to bind to and block the Wnt coreceptor LRP5/6 [11,12]. A similar mechanism was shown for the four members (Dkk1–4) of the Wnt modulator family dickkopf, which share no sequence similarity with sclerostin and also block Wnt receptor activation by binding to LRP5/6 [23]. Sclerostin's negative impact on bone formation is also seen from targeted deletion of *SOST* in mice [24]. Sclerostin knockout mice display a strongly increased bone formation in the limb and massively enhanced bone strength [24]. Interestingly, the increase of bone formation was limited to the skeleton and no ectopic bone formation was observed. These properties make sclerostin a highly interesting drug target for a new osteoanabolic treatment of osteoporosis, as can be seen from current attempts to bring an anti-sclerostin drug to the market ([25,26], for review, see [9]).

Sclerostin shares limited sequence similarities with the bone morphogenetic protein (BMP) modulator proteins of the DAN family [27]. DAN members as well as sclerostin contain a cystine-knot motif, which comprises six cysteine residues forming a knot from three disulfide bonds; however, sclerostin and the related WISE (SOSTDC1) were shown to be monomeric proteins [28–30] and the classical DAN members such as gremlin, PRDC (gremlin2) and NBL1 seem to function as homodimers ([31,32], for review, see [33]). Furthermore, whereas classical DAN members indeed impede BMP signalling by binding BMPs with high affinity [34], sclerostin was shown to act on the Wnt pathway and not by blocking BMP receptor activation [35]. The different architecture is also reflected in structural differences. The DAN members NBL1 and PRDC exhibit an arc-like dimer structure, in which all three loops emanating from the cystine-knot core are highly structured. In sclerostin, only the first and the third loops, which are running in parallel from the central cystine-knot, are structured forming two 2-stranded β-sheets, termed fingers 1 and 2 [29,30]. The second loop, which runs in the opposite direction, is highly flexible due to lack of structure-forming van der Waals contacts, as are present in the dimer interface of the DAN members PRDC and NBL1. Interestingly, several studies indicate that this flexible loop is important for sclerostin's ability to neutralize Wnt signalling. First, Veverka *et al*. [29] showed that an antibody neutralizing sclerostin's inhibitory activity on Wnt/β-catenin signalling binds to the flexible second loop. Second, structure–function studies showed that mutations in the tip of that loop impair binding of sclerostin to LRP6 as well as its Wnt inhibitory capacity [36,37].

The Wnt co-receptors LRP5 and LRP6 are modular proteins comprising four propeller domains in the extracellular part. Li *et al*. [11] were able to show that binding of sclerostin to LRP5 only requires the first two propeller domains, whereas the Wnt modulator Dkk1 interacts with all four propeller domains of LRP5 indicating that the inhibitory mechanism of sclerostin and dickkopf proteins possibly differs. Structure–function studies then confirmed that the C-terminal domain of Dkk1 binds to propeller 3 and 4 of LRP6 [38–40], whereas a short tri-peptide segment (NXI) present in the N-terminal segment of Dkk1 as well as in the above-mentioned second loop of sclerostin and WISE (SOSTDC1) binds to the first propeller domain of LRP6 [41]. Thus, Wnt inhibition by Dkk1 and sclerostin seems to follow a competition mechanism by which binding of the modulator proteins blocks the interaction of Wnt factors with LRP5/6.

For further studies on how sclerostin antagonizes Wnt signalling and which determinants are involved in Wnt inhibition, we have developed antibodies against sclerostin employing a phage-selection procedure. Using recombinant human and murine sclerostin proteins as well as loop-mimicking peptides as antigens, we obtained various Fab antibody fragments showing high binding specificity for sclerostin. Functional analyses such as surface plasmon resonance (SPR), peptide mapping and cellular assays were used to characterize *in vitro* properties, providing a ‘tool set’ comprising species-specific Fabs as well as different antibodies that bind virtually to any region of sclerostin. Furthermore, an antibody AbD09097 was obtained that neutralizes sclerostin's ability to inhibit Wnt signalling. To further improve its efficiency, we applied affinity maturation to this Fab fragment. A crystal structure analysis of AbD09097 provides the first high-resolution structural insights into a neutralizing anti-sclerostin antibody, which will certainly facilitate new approaches for therapies targeting osteoporosis.

## 2. Material and methods

### 2.1. Protein production

For developing anti-sclerostin antibodies via a phage-panning selection, recombinant human and murine sclerostin were expressed in Sf9 insect cells as full-length proteins containing an N-terminal hexahistidine-tag followed by a thrombin cleavage site as published [30]. Proteins were isolated employing metal-chelate affinity chromatography using Ni^2+^-NTA as resin (Qiagen) and a subsequent cation-exchange chromatography using CM Sepharose (GE Healthcare) and a linear gradient of 0–1 M sodium chloride in 10 mM HEPES pH 7.5.

Human and murine sclerostin proteins and variants thereof used for *in vitro* interaction analysis and cellular assays such as reporter gene studies were expressed, refolded and purified from *Escherichia coli* as published previously [30,42].

^15^N-labelled murine sclerostin used for nuclear magnetic resonance (NMR) chemical shift titration mapping to determine the binding sites of Fab antibodies was produced in *E. coli* Rosetta (DE3) using M9 minimal medium supplemented with 0.5 g l^−1^ ^15^NH_4_Cl as described [30]. After refolding of the protein the N-terminal hexahistidine-tag was removed using biotinylated thrombin (Novagen). The endopeptidase was removed by streptavidin agarose (Novagen). Highly pure ^15^N-labelled murine sclerostin protein was then obtained by applying RP-HPLC using a preparative C8 column and employing a water–acetonitrile gradient in 0.1% (v/v) trifluoroacetic acid.

### 2.2. Peptide production

Peptides were synthesized by automated Fmoc-based solid-phase peptide synthesis using a Rink-amide resin (Bachem) on a Symphony peptide-synthesizer (Protein Technologies). Crude peptides were purified by C18 RP-HPLC. The correct molecular masses of the peptides were confirmed by electro-spray ionization mass spectrometry on an Aquity™ SQD mass spectrometer (Waters).

The cysteine residues on peptide PFD038, comprising residues 86–111 of human sclerostin, were either cross-linked with 1,3-bis(bromomethyl)benzene (mT2 CLIPS^TM^) (PFD038_mT2), disulfide-bridged by oxidation (PFD038_ox), or reacted with iodoacetamide (PFD038_IAM) to enforce linear peptide structure [42,43].

### 2.3. Phage display library panning and Fab generation

For generation of antibodies against murine and human sclerostin the HuCAL GOLD library [44] was used. Sclerostin proteins were biotinylated using EZ-link Sulfo-NHS-LC-Biotin (Thermo Fisher Scientific), coupled to streptavidin-coated magnetic beads (Dynal, Thermo Fisher Scientific) and antigen-binding antibodies were selected with these sclerostin-coated beads by three rounds of solution panning. In each panning round, the antibody phage library was incubated with biotinylated sclerostin protein, binding Fabs were captured via streptavidin-coated magnetic beads (Invitrogen) and phages were eluted with 25 mM DTT. To obtain antibodies specific for the target protein, the beads were washed at room temperature on a Kingfisher instrument (Thermo Fisher Scientific). The first round of washing was performed six times for 1 min, in the second round a more stringent washing was done eight times for 1 min 30 s, and in the third round the beads were washed eight times for 3 min with PBS buffer containing 0.05% (v/v) Tween 20 (PBST), followed in each round by one wash cycle in PBS for 2 min. To obtain species-specific antibodies against murine and human sclerostin, separate rounds of panning were performed on both recombinant proteins.

For screening of the antibodies, *E. coli* of the strain TG1F^−^ (TG1 without the F-plasmid) were transformed and individual colonies were randomly picked and grown in micro-titre plates. After expression overnight at 22°C upon induction with 1 mM IPTG, the cultures were chemically lysed (0.4 M boric acid buffer, pH 8.0 containing 320 mM NaCl, 4 mM EDTA, 0.25% (w/v) lysozyme and 12.5 U ml^−1^ Benzonase^®^ (Merck Millipore)) and the crude extracts were tested via ELISA for the presence of specifically binding antibody fragments. For all clones exhibiting a strong binding signal for the antigens (greater than or equal to fivefold over background) the DNA sequence encoding the complementarity-determining regions (CDRs) of the antibody variable heavy chain was determined. Colonies containing antibodies with unique CDR3 sequence were chosen for subsequent purification.

After three rounds of panning, the pool of Fab genes was isolated and inserted into *E. coli* expression vectors. These vectors allow for functional periplasmic expression of monovalent Fab equipped with two peptide sequences at the C-terminus of the antibody heavy chain, a myc-tag (EQKLISEEDL) and a hexahistidine-tag, the latter of which was used for purification. This format (called Fab-Thr-MH) also contains a cleavage site between the CH1 domain and the tag region for the endopeptidase thrombin allowing for proteolytic removal of both sequences. Preparative production of recombinant Fabs was performed as described [45].

### 2.4. Affinity maturation by targeted diversification of the CDR3 of the variable light chain

The DNA fragment encoding the Fab AbD09097 was subcloned into a phagemid vector based on pMORPH23 [24,44]. For affinity maturation, a phage library was generated in which the DNA region encoding the variable light chain CDR3 was replaced with a repertoire of variable light chain CDR3 sequences. *Escherichia coli* TOP10F’ cells (Invitrogen) were transformed with the ligated vectors generating a library of about 3 × 10^6^ different clones. This phage library was subjected to a solution panning approach as described above but using decreasing amounts of biotinylated recombinant human sclerostin (produced from Sf9 insect cells) in the two selection rounds (230 nM biotinylated recombinant human sclerostin in the first and 23 nM in the second round of panning). Compared with the panning procedure for generation of the Fab antibodies above a more stringent washing was used. In the first round of maturation washing was eight times for 3 min with PBST and in the second round 24 times for 3 min with PBST, followed in each round by one wash cycle in PBS for 2 min. Selection and production of the affinity-maturated Fab antibodies was performed as described above.

### 2.5. ELISA for selection of specific anti-sclerostin antibodies

Black Nunc MaxiSorp 384 well plates (Thermo Fisher Scientific) were coated overnight at 4°C with a 5 µg ml^−1^ solution of protein in PBS. For immobilization of the biotinylated peptides and proteins, neutravidin (Pierce) was first coated in the wells. After blocking non-specific binding sites with 5% (w/v) bovine serum albumin in PBST, the biotinylated antigens were added to the wells at a concentration of 2 µg ml^−1^ and the plates were incubated for 30 min at room temperature. Then the microtitre plates were washed and an aliquot of the Fab protein at a concentration of 2 µg ml^−1^ was added to each well. For binding of the Fab to the presented antigens the plates were incubated for one hour at room temperature. Detection was performed using an anti-human Fab–alkaline phosphatase conjugate (AbD Serotec) using AttoPhos (Roche) as a substrate. Signals from this Fab–secondary detection antibody pair interacting with immobilized control proteins BSA, N1-CD33-His6 (the ectodomain of human CD33 fused to the N1 domain of the g3p filamentous phage M13 as described in [46]) and glutathione-*S*-transferase (GST) were used for calculation of the background.

### 2.6. Interaction analysis using surface plasmon resonance

The SPR analyses for the measurement of *in vitro* interactions were performed using a ProteOn^TM^ system (Bio-Rad) at a temperature of 25°C as described [42]. HBS150T (10 mM HEPES, 150 mM NaCl, pH 7.5, 0.005% (v/v) Tween 20) was used as running buffer. Murine or human sclerostin proteins or variants thereof were used as ligands. They were immobilized to a surface density of about 600 resonance units (RU) on an activated ProteOn^TM^ GLC sensor chip (Bio-Rad) in vertical direction using amine coupling. The antibodies were used as analytes and were injected simultaneously in six different concentrations (100, 75, 50, 25, 12.5 and 6.25 nM in HBS150T unless indicated otherwise) in horizontal direction (single-shot kinetic set-up). The association was monitored for 200 s at a flow rate of 100 µl min^−1^. Data for dissociation were acquired for 200 s by perfusing HBS150T buffer at a flow rate of 100 µl min^−1^. Unspecific binding and bulk face effects were removed by subtracting the interaction of the analyte with a non-modified surface of a control flow channel. The chip surface was regenerated by a 60 s pulse of 10 mM glycine pH 2.0. Binding affinities were determined from the rate constants for association and dissociation using the ProteOn^TM^ Manager 3.1 software (Bio-Rad) and applying a simple 1 : 1 Langmuir type interaction model. The binding response was normalized by determining the maximal binding response (Rmax) using the interaction data of all antibody proteins measured and then applying this maximal value in the fitting of the data to obtain the association rate constant.

### 2.7. Wnt reporter gene assay

Cells were cultivated at 37°C and 5% CO_2_ in DMEM (Invitrogen) containing 10% (v/v) FCS, 100 U ml^−1^ penicillin G, 100 µg ml^−1^ streptomycin (Invitrogen). A stable reporter cell line (cell pool) termed HEK293TSA M50 for quantitative measurement of Wnt activity was obtained by transfection with the reporter construct M50 Super 8× TopFlash (provided by Randall Moon) and a linearized hygromycin marker DNA (Clontech) as described previously [36]. This cell pool shows a dose-dependent luciferase expression in response to exogenously applied recombinant Wnt3a or transfection of the cells with a Wnt3a or Wnt1 expression plasmid.

Wnt reporter gene assays were performed as described [36]. Briefly, cells were seeded in 10 cm cell culture dishes (1.5 × 10^5^ cells ml^−1^), transfected the next day with 12 µg mWnt1 expression vector (mouse cDNA clone MC205633, Origene) or the same amount of empty vector DNA (pEF6B, Invitrogen) and transferred to 96-well plates the following day; 48 h post-transfection cells were incubated with different concentrations (0–100 nM) of wild-type murine or human sclerostin alone or in the presence of indicated Fab fragments (500 nM). In a different set-up, cells were incubated using a constant sclerostin concentration (20 nM) and varying concentrations of Fab fragments. In each case, stimulated cells were lysed after 24 h using 50 µl reporter lysis buffer (Promega) per well and one freeze and thaw cycle at −80°C. Twenty microlitres of the obtained cell lysate were mixed with 20 µl of luciferase assay substrate (Promega) and luciferase activities were measured (Luminoscan Ascent, Labsystems). GraphPad Prism was used for analysing the data by nonlinear regression (dose response inhibition or dose response stimulation function).

### 2.8. Nuclear magnetic resonance mapping of the Fab-binding epitope on sclerostin

About 25 nmol of freeze-dried ^15^N-labelled sclerostin variant SOSTΔNC (truncated murine sclerostin comprising residue N36–R144) was dissolved in NMR buffer (20 mM potassium phosphate pH 6.0, 50 mM NaCl, 5% (v/v) D_2_O, 0.2% (w/v) NaN_3_). A second NMR sample contained 25 nmol ^15^N-labelled SOSTΔNC and additionally 25 nmol of the Fab AbD09097 (unlabelled). To ensure identical buffer conditions, both samples were dialysed against the same NMR buffer, subsequently concentrated to 250 µl (Centricon, Millipore) and transferred into Shigemi tubes (Shigemi Corp.). NMR spectra were measured at 27°C using a 600 MHz Bruker Advance spectrometer equipped with a triple-resonance, triple axis cryoprobe. Data processing and analysis was performed using the software TopSpin 2.0 and Aurelia (Bruker, Rheinstetten).

### 2.9. Epitope mapping of the Fab antibodies by peptide arrays

To map the sclerostin epitopes recognized by the Fab antibodies obtained from panning, peptide libraries were synthesized on solid support [47]. Libraries consisted of all overlapping linear and CLIPS constrained cyclic 15mer peptides derived from murine and human sclerostin. In addition, libraries of all overlapping linear 4, 5, 6, through to the 24mer peptides mimicking the flexible loop 2 of human sclerostin were prepared. For the peptide replacement array a series of substitution mutants of the best binding 14mer (PARLLPNAIGRGKW) were prepared, in which each residue was exchanged for all other proteinogenic amino acids except Ile. Similarly two peptide libraries for a truncation array analysis were synthesized on the basis of two 14mer peptides (PARLLPNAIGRGKW and NAIGRGKWWRPSGP) consisting of all truncated peptide variants from 3 to 14mer length. After titration with the antibodies, a modified ELISA using a goat anti-human peroxidase coupled secondary antibody was carried out as described [48].

### 2.10. Crystallization and structure determination of the sclerostin-neutralizing Fab AbD09097

For crystallization, the Fab AbD09097 was further purified by cation-exchange chromatography employing SP-Sepharose (GE Healthcare) and a linear gradient from 0 to 1 M NaCl in 50 mM sodium acetate pH 5.0. Fractions containing pure Fab protein were pooled, dialysed against 10 mM Tris–HCl pH 7.6, 50 mM NaCl and concentrated to about 10 mg ml^−1^ using ultrafiltration.

Initial crystallization trials were performed using commercially available screens (Qiagen/Nextal PACT, PEGs I and II suites) using a sitting drop vapour-diffusion set-up. One microlitre protein solution (7.5–10 mg ml^−1^) was mixed with 1 µl reservoir solution and placed above 100 µl reservoir solution. Optimization and production of crystals for data acquisition were performed using a hanging drop vapour-diffusion set-up. Crystals of AbD09097 suitable for diffraction data acquisition were grown from 20% (w/v) PEG3350, 100 mM HEPES pH 7.5, 10 mM ZnCl_2_. Rod-shaped crystals with dimensions of 200 × 50 × 50 µm^3^ grew within three weeks. Diffraction data from a single crystal at 100 K were measured using an X-ray home source, a Rigaku MicroMax-007 HF X-ray generator equipped with VariMax HF mirror optics and a Rigaku R-AXIS HTC image plate detector system. Data were processed and analysed using the software iMosflm and CCP4i. Structure analysis and model building was performed using the software Quanta2008 (MSI Accelrys, San Diego). For details of crystallization, data acquisition and processing, see Boschert *et al.* [49] (for data statistics, see electronic supplementary material, table S1).

## 3. Results

### 3.1. Generation of anti-sclerostin antibody fragments (Fabs) using recombinant sclerostin as antigen

**Figure 1.**
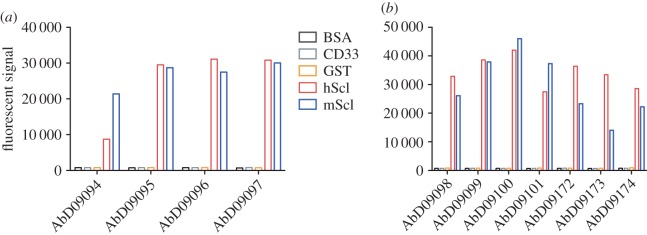
Specificity of Fabs derived from phage display using murine (*a*) and human (*b*) sclerostin as antigen. An ELISA was used to determine binding of the selected Fabs to murine and human sclerostin (mScl and hScl) and control proteins bovine serum albumin (BSA), human CD33 and glutathione-*S*-transferase (GST). Shown is a single experiment; binding characteristics were then further analysed using SPR (figures 2 and 3; table 1).

**Figure 2.**
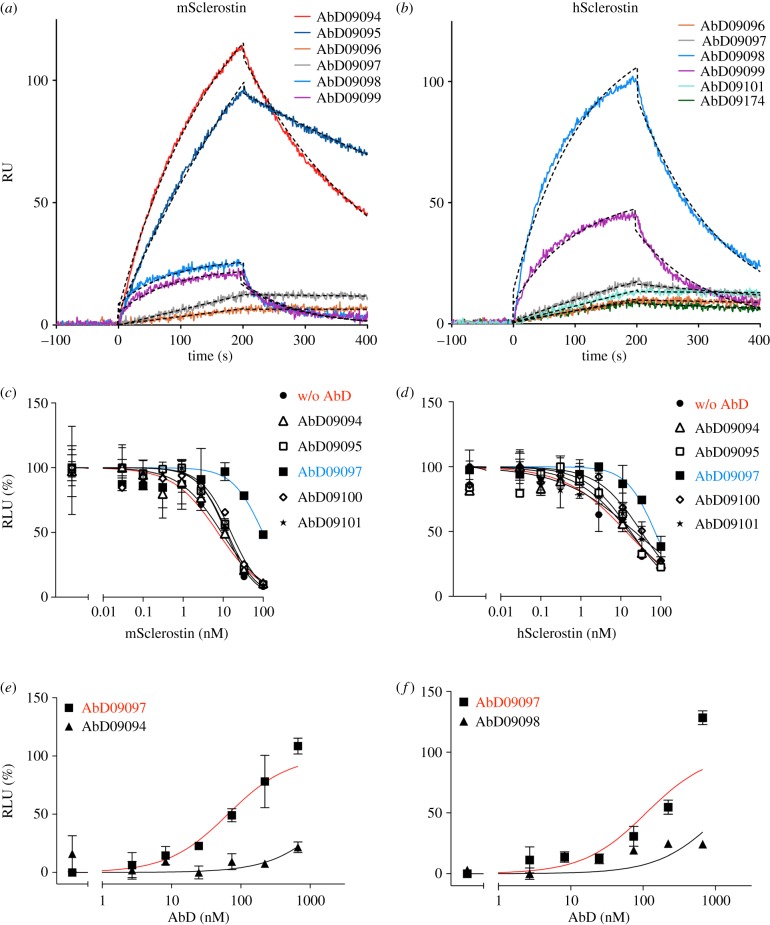
SPR analysis for the interaction of the Fab proteins with murine (*a*) and human (*b*) sclerostin. Sclerostin proteins were immobilized as ligands on the biosensor at a density of about 600 RU using amino coupling chemistry. At time point zero Fab proteins were injected using six different analyte concentrations and the association was measured for 200 s. Thereafter, buffer was perfused over the biosensor for 200 s to acquire the data for dissociation. For a direct comparison of the rate constants an overlay of the sensograms of selected Fab proteins at a single analyte concentration of 25 nM is shown. Fitted data are shown as black dashed lines. To determine the effect of antibody binding on sclerostin, HEK293TSA M50 cells were transfected with an expression vector for mWnt1 and incubated with serial dilutions of murine (*c*) or human (*d*) recombinant sclerostin with or without the indicated Fab fragment (500 nM). The Wnt1-mediated luciferase expression was analysed and the concentration of sclerostin is plotted against relative luciferase units (RLU) in percentage of RLU obtained with Wnt1-transfected but untreated cells (first data point). In a similar experimental set-up, the transfected cells were incubated with serial dilutions of the indicated antibody fragment in the presence of a constant concentration (20 nM) of murine (*e*) or human (*f*) sclerostin. The signal obtained with sclerostin alone (first data point) was set to 0% and the signal of Wnt1-transfected but untreated cells was set to 100%. Data points represent means of two values, error bars indicate s.d. Shown are typical experiments out of three.

The Fab phage display library HuCAL GOLD was used for generation of anti-sclerostin antibodies, employing recombinant murine or human full-length sclerostin as antigen. For each selection, 368 clones were tested for binding to the antigen. From the panning employing murine sclerostin four unique Fabs (designated AbD09094, 09095, 09096 and 09097) could be obtained, whereas panning using human sclerostin yielded seven unique Fabs (designated AbD09098, 09099, 09100, 09101, 09172, 09173 and 09174). These 11 Fab fragments were expressed on a larger scale, purified and their binding properties were tested in an ELISA using murine and human sclerostin as well as unrelated control proteins to reveal their specificity (figure 1). Specific binding could be confirmed for all 11 antibodies. One Fab from the panning on murine sclerostin (AbD09094) showed preferential binding to the murine antigen and three Fabs from the panning on human sclerostin (AbD09172, 09173 and 09174), a better binding on the human antigen. For more detailed information on the binding properties of the various sclerostin-targeting Fabs, we determined the binding kinetics using SPR. Owing to the sticky nature as apparent from unspecific interaction with the sensor matrix, human and murine sclerostin were used as ligands immobilized on the chip surface. The (monovalent) Fab antibodies were used as analytes and the binding kinetics were determined employing the so-called one-shot kinetics set-up, i.e. the analyte was perfused over the biosensor simultaneously in six different concentrations ranging from 6.25 to 100 nM (figure 2*a*,*b*; table 1). From the antibodies against murine sclerostin, Fabs AbD09094, 09095 and 09097 exhibited a high affinity (between 50 and 60 nM) specifically for murine sclerostin, but either did not bind human sclerostin at all or did so with at least fivefold lower affinity (table 1). Similarly, from the antibodies derived from panning using human sclerostin, the Fabs AbD09101 and AbD09172 bound the human antigen with high affinity (AbD09101, *K*_D_ = 42 nM and AbD09172, *K*_D_ = 12 nM) and did not interact with the murine homologue at all. The Fab AbD09096 bound both isoforms similarly with a *K*_D_ of 199 nM for human and a *K*_D_ of 157 nM for murine sclerostin, although the antibody was obtained from a panning employing murine sclerostin. The five remaining antibodies, which originate from the selection on human sclerostin, bind only to the human protein, however, their binding affinities are rather low with *K*_D_ values between 200 and 960 nM (table 1). In summary, we successfully obtained 11 different Fab antibodies from phage display employing full-length murine and human sclerostin, of which five recognize native sclerostin with affinities in the nanomolar range in a species-specific manner and thus represent valuable tools for detection of sclerostin by various methods.

**Table 1.** Fab antibodies against murine and human sclerostin. Analysis of Fab antibodies derived from phage display using murine and human sclerostin as antigens. The binding affinities of these Fabs were determined using surface plasmon resonance (SPR), *K*_D_ values and kinetic parameters were obtained using a set-up with the Fab antibody proteins as analytes (six different concentrations) and human and murine sclerostin as immobilized ligand. N.B. indicates no binding in the SPR experiment, which under the measurement conditions corresponds to a lower binding affinity boundary of less than 5 µM; n.d., not determined. Changes in bioactivity were measured by a Wnt-responsive reporter gene assay using HEK293TSA cells transfected with Wnt1. Indicated are the changes in IC_50_ value upon addition of Fab antibody compared with cells treated with recombinant sclerostin proteins alone (means of at least two experiments).

antigen	AbD	SPR						reporter assay	
		murine sclerostin			human sclerostin			fold change IC_50_	
		*k*_on_ (×10^3^ M^−1^ s^−1^)	*k*_off_ (×10^−4^ s^−1^)	*K*_D_ (nM)	*k*_on_ (×10^3^ M^−1^ s^−1^)	k_off_ (×10^−4^ s^−1^)	*K*_D_ (nM)	mScl.	hScl.
murine sclerostin	AbD09094	65	39	61^a^	—	—	N.B.	1.2	1.5
	AbD09095	34	17	53	5.8	94	≥1000	1.2	1.4
	AbD09096	2.1	2.7	157	3.5	6.7	199	2.3	1.3
	AbD09097	4.4	1.8	46	6.6	16	260	12.6	6.4
human sclerostin	AbD09098	1.1	96	881	3.5	6.6	193	0.9	1.0
	AbD09099	1.1	96	935	31	90	307	1.1	1.5
	AbD09100	—	—	N.B.	35	390	964	2.9	3.6
	AbD09101	—	—	N.B.	4.5	1.7	42	1.9	3.7
	AbD09172	—	—	N.B.	150	16	12^a^	0.8	0.8
	AbD09173	—	—	n.d.	—	—	n.d.	n.d.	n.d.
	AbD09174	—	—	N.B.	340	13	394	1.2	1.4

^a^Marks SPR analyses with *χ*^2^ > 10% of the maximal response value indicating that the applied simple Langmuir 1 : 1 interaction type model does not sufficiently fit the experimental binding kinetics.

### 3.2. The Fab AbD09097 neutralizes sclerostin's ability to inhibit Wnt activity

The antibodies were then tested for their effect on sclerostin bioactivity employing a reporter gene assay and using a HEK293 cell line stably expressing a Wnt-responsible luciferase reporter construct. For stimulation of the Wnt/β-catenin pathway the cells were first transfected with a plasmid encoding for Wnt1. Two days after transfection recombinant sclerostin was added to the cells resulting in a dose-dependent inhibition of the Wnt1-mediated signal. The rescue from sclerostin-mediated Wnt1 inhibition was then measured by adding the Fab antibodies in a concentration of 500 nM to different sclerostin concentrations (0–100 nM) and is shown as fold change of sclerostin's IC_50_ for the inhibition of Wnt1 activity (figure 2*c*,*d*). As shown in table 1, most Fabs (AbD09094, 09095, 09096, 09098, 09099, 09172 and 09174) did not significantly affect sclerostin's ability to neutralize Wnt/β-catenin signalling.

For three Fabs, AbD09097, 09100 and 09101, the IC_50_ value for sclerostin-neutralizing Wnt1 activity was decreased in the presence of these antibodies indicating that these Fabs would interfere with sclerostin's ability to compete with Wnt1 for binding to the Wnt co-receptor LRP5/6. This effect was, however, quite small for AbD09100 and AbD09101 (two- to fourfold change in the IC_50_ values). Furthermore, the changes in the IC_50_ values were similar for human and murine sclerostin, even though both antibodies in SPR specifically bound only human sclerostin, which seriously questions the specificity of the neutralizing effect of these two Fab antibodies. By contrast, Fab AbD09097 neutralized sclerostin-mediated inhibition of Wnt1 activity with high efficiency. The concentration for half-maximal inhibition of Wnt1 by murine sclerostin was on average shifted almost 13-fold in the presence of AbD09097 and sixfold when human sclerostin was used to inhibit Wnt1 activity (table 1 and figure 2*c*,*d*). Performing a Wnt1 reporter gene assay with a constant concentration for both sclerostin proteins of 20 nM and different concentrations of AbD09097 (0–670 nM) confirmed the better efficacy in inhibiting murine sclerostin (figure 2*e*,*f*). The Fab showed an effective concentration (EC_50_) of 46 ± 27 nM (*n* = 4) for murine sclerostin and a fourfold lower activity (EC_50_ of 197 ± 123 nM (*n* = 3)) for human sclerostin. This difference in efficacy might be explained by the binding properties of AbD09097 towards both sclerostin proteins (table 1).

### 3.3. Sclerostin-neutralizing antibodies bind to sclerostin's flexible loop

**Figure 3.**
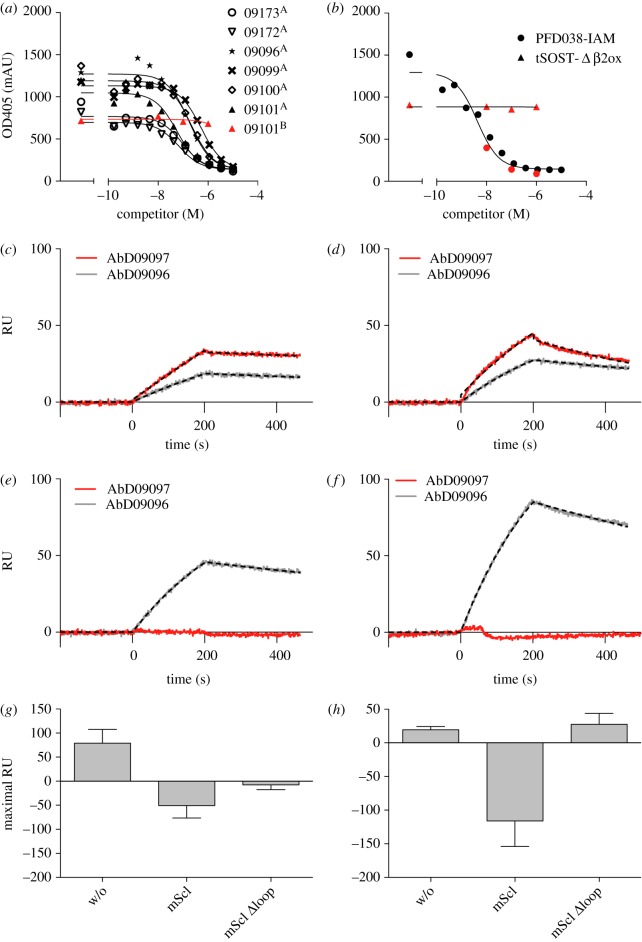
AbD09097 binds to sclerostin's flexible loop. (*a*) Competition ELISA measuring the binding of selected Fab antibodies to immobilized sclerostin (human form, for AbD09096 and AbD09099 murine sclerostin) in the presence of the peptide tSOST-Δβ2ox (marked with A), which resembles a truncated sclerostin protein only comprising finger 1 and 2, and PFD-038IAM (marked with B and displayed in red), which mimics the linear form of sclerostin loop 2. The data are derived from a single experiment. (*b*) Competition ELISA measuring the binding of the Fab antibody AbD09097 to immobilized murine sclerostin in the presence of an increasing concentration of the peptides PFD-038IAM and tSOST-Δβ2ox. The binding of the Fab was determined by a secondary anti-Fab alkaline-phosphatase conjugate. The red and black data points indicate two separate experiments combined in one graph. (*c*–*f*) SPR sensograms are shown for the interaction of AbD09097 and AbD09096 with murine (mScl, *c*), human (hScl, *d*) and the murine sclerostin variants Δloop (*e*) and AlaLoop (*f*). The sclerostin proteins were immobilized on the chip surface. At time point zero AbD09097 was injected as analyte using six different concentrations (100, 75, 50, 25, 12.5 and 6.25 nM), curves for 75 nM are shown. At time point 200 s injection of the Fab protein was stopped and the chip surface was perfused with buffer. Fitted curves are shown as dashed lines. (*g*–*h*) SPR competition experiment for the Fabs AbD09096 (*g*) and AbD09097 (*h*). The left bars (marked with w/o) show the signal (in resonance unit, RU) measured at 200 s perfusion of 300 nM Fab fragment over a biosensor coated with 600 RU wild-type murine sclerostin. The middle bars (marked with mScl) show the signal obtained when 300 nM of the respective Fab antibody was perfused in the presence of 600 nM wild-type murine sclerostin. As sclerostin shows unspecific binding to the chip surface, the signal from perfusing 600 nM sclerostin without any Fab protein was subtracted from the raw signal obtained for co-injection of Fab and sclerostin. The right bars (marked with mScl Δloop) show the signal after co-injecting 300 nM Fab in the presence of 600 nM of the variant Scl Δloop, which lacks loop 2. Data processing included subtraction of an injection with 600 nM Scl Δloop to remove the effect of unspecific binding of sclerostin to the chip surface as stated above. Shown are mean RU values with s.d. from at least eight experiments. Means of the binding signal for AbD09096 alone and co-injected with sclerostin or the variant mScl Δloop differ significantly (*p* < 0.0001), just as do the means of the binding signal for AbD09097 alone and co-injected with murine sclerostin (*p* < 0.0001). Means of AbD09097 alone and co-injected with the variant mScl Δloop do not differ significantly (*p* = 0.1166). Statistical results were obtained with an unpaired two-tailed Student's *t*-test.

As the strong neutralizing capability of AbD09097 is a unique property not seen for the other antibodies, we tried to determine the binding epitope of this Fab. There are reports of other sclerostin-neutralizing antibodies, which have already been tested in clinical trials [25,26]. Only for one has the binding epitope on sclerostin been investigated. Using NMR it was shown that the antibody Scl-AbI binds in the flexible second loop of human sclerostin [29]. This finding seems consistent with the notion that the second loop, namely the loop tip containing the NXI-motif, is the main binding determinant for binding of sclerostin to the first propeller of LRP6 [36,37,41]. In order to determine the part of sclerostin recognized by AbD09097, we initially performed a competitive ELISA (figure 3*a*,*b*) using structured peptides corresponding to defined regions of sclerostin as competitors. The ELISA plate was coated with recombinant sclerostin and then probed with the different Fabs. Two different peptides, PFD038-IAM and tSOST-Δβ2ox, mimicking different sclerostin regions were used to compete for the antibody binding. tSOST-Δβ2ox is a native cysteine-bridged construct of the loops 1 and 3, but lacks loop 2 [42]. PFD038-IAM comprises loop 2 and the cysteines at the termini are carboxyamidomethylated to enforce a linear peptide form. The non-neutralizing Fabs AbD09096, 09099, 09100, 09101, 09172 and 09173 could be displaced with the peptide tSOST-Δβ2ox suggesting that their binding site is outside loop 2 (EC_50_ from 68 to 242 nM) (figure 3*a*). However, AbD09097 was the only antibody that could be displaced with the loop 2-mimicking peptide PFD038-IAM (EC_50_ = 6.4 nM), indicating that the neutralizing property of AbD09097 is possibly linked to its binding epitope located in the flexible loop 2 of sclerostin (figure 3*b*). To confirm this finding, we performed an SPR analysis using murine and human sclerostin and two variants thereof (figure 3*c*–*f*), Scl Δloop and Scl Alaloop, in which loop 2 is either truncated (in the variant Scl Δloop Leu90 to Asn103 are replaced by a short glycine–serine linker) or replaced by a scrambled amino acid sequence (Scl Alaloop) and which were shown to be strongly impaired in blocking Wnt1 activity [36]. No binding of AbD09097 could be observed to both sclerostin variants (figure 3*e*,*f*; electronic supplementary material, table S2). To further corroborate this hypothesis we performed SPR competition analyses. Here the neutralizing AbD09097 or exemplarily the non-neutralizing AbD09096 were perfused over a biosensor coated with sclerostin either alone or in the presence of a twofold excess of murine sclerostin or the variant Scl Δloop. For the Fab AbD09096, wild-type sclerostin as well as the variant lacking loop 2 (Scl Δloop) could abrogate the binding of AbD09096 to the sclerostin immobilized on the sensor chip surface (figure 3*g*). This clearly shows that the non-neutralizing AbD09096 recognizes and binds an epitope outside loop 2. By contrast, binding of the Fab AbD09097 to the sclerostin biosensor was only impeded when wild-type full-length sclerostin was co-injected. In the presence of the variant Scl Δloop, the neutralizing Fab AbD09097 could still bind to the sclerostin SPR sensor indicating that the sclerostin variant lacking loop 2 was incapable of blocking this interaction (figure 3*h*). Together ELISA and SPR results imply that the binding epitope of AbD09097 is restricted to the flexible loop 2 of sclerostin and that the location of the epitope is linked to its neutralizing activity against sclerostin-mediated Wnt inhibition.

### 3.4. Nuclear magnetic resonance-based epitope mapping of the sclerostin-neutralizing Fab AbD09097

**Figure 4.**
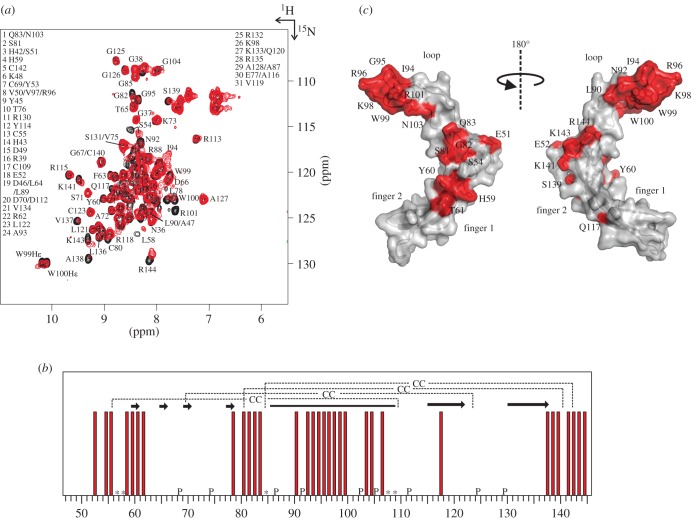
NMR mapping study to determine the binding epitope of the neutralizing Fab AbD09097. (*a*) Overlay of ^1^H^15^N 2D HSQC spectra of the uniformly ^15^N-labelled murine sclerostin variant ΔNC in the absence (black) and presence (red) of an equimolar amount of AbD09097. NMR signals of sclerostin affected by the binding of AbD09097 (i.e. visible either from changes in the chemical shift or from altered line width) are indicated. (*b*) Bar diagram highlighting the residues involved in binding of AbD09097 as observed by NMR mapping. P denotes proline residues which do not have an amide nitrogen–proton correlation, asterisk (*) denotes residues whose correlation signal could not be assigned unambiguously. (*c*) Van der Waal surface representation of sclerostin with the amino acid residues whose NMR signals were affected upon binding of AbD09097 highlighted in red. In the right panel, the sclerostin structure is rotated around the *y*-axis by 90°.

To precisely identify the residues of sclerostin contributing to the binding of the neutralizing Fab, we performed NMR chemical shift mapping analysis by measuring ^1^H^15^N 2D-HSQC spectra of uniformly labelled ^15^N-labelled murine sclerostin lacking the flexible N- and C-terminus (SclΔNC [30]) in the presence and absence of AbD09097 (figure 4). Both proteins were present at the same concentration and as the binding of AbD09097 to sclerostin is strong (table 1) and dissociation of the complex is very slow (see also figures 2*a* and 3*c*), a correlation signal distinct from that of free sclerostin is to be expected for each residue of bound sclerostin which is in close proximity to the antibody binding site.

Of note, the ^1^H^15^N 2D HSQC experiment of the sclerostin–antibody complex yielded a spectrum with most signals exhibiting a line width comparable with that in the spectrum of free sclerostin. That was unexpected as usually NMR signals get somewhat broadened due to the slow overall tumbling rate and the subsequent fast relaxation of the magnetization for proteins/protein complexes of this size, which can be overcome by applying special techniques such as TROSY or deuteration [50,51]. This, however, is mainly true for proteins not exhibiting strong internal dynamics, which is not the case for sclerostin. There it has been shown that loop 2 is rather flexible as indicated by hetero-nuclear NOEs [30]. Consequently, the lines of those residues are broadened by exchange in the free form of sclerostin. Binding to AbD09097 would then reduce the dynamics and sharpen the lines which counteracts the effect of line broadening due to the overall size of the complex. The finger regions are not in contact with the antibody and due to the internal flexibility retain their original correlation time and line width.

When compared with the spectrum of free sclerostin, chemical shift differences for a defined subset of amino acid residues were observed indicating specific binding of the Fab to sclerostin. A more detailed analysis indicated that the chemical shifts of several NMR signals were altered or broadened in the spectrum of sclerostin bound to AbD09097 (figure 4*a*,*b*). A graphical representation suggests that the binding epitope of AbD09097 is located at the tip of loop 2 of sclerostin comprising residues Leu90 to Gly104, but that the binding site of the Fab also either includes residues close to the cystine-knot (e.g. residues Glu52 to Tyr60, Leu78 to Gln83 and Val137 to Arg144) or affects their conformation otherwise (figure 4*b*,*c*). Thus, the neutralizing Fab AbD09097 has a very similar binding epitope to that observed for the neutralizing anti-sclerostin antibody studied by Veverka *et al*. [29]. In the latter antibody, the chemical shift values of residues Ala89 to Asp108 of human sclerostin (equivalent to Ala87 to Asp106 in murine sclerostin) located in the flexible loop 2 showed the highest difference between free and bound sclerostin. Similar to our findings also some residues in or close to the cystine-knot (e.g. Ala140 to Cys144) seemed to be affected or part of the antibody binding site, which might be explained by the fact that the size of an antibody epitope may consist of up to 20 amino acid residues and thus the tip of loop 2 may be too small to cover all of the antibody binding site.

### 3.5. Determining the main binding determinant for the sclerostin–Fab interactions

**Figure 5.**
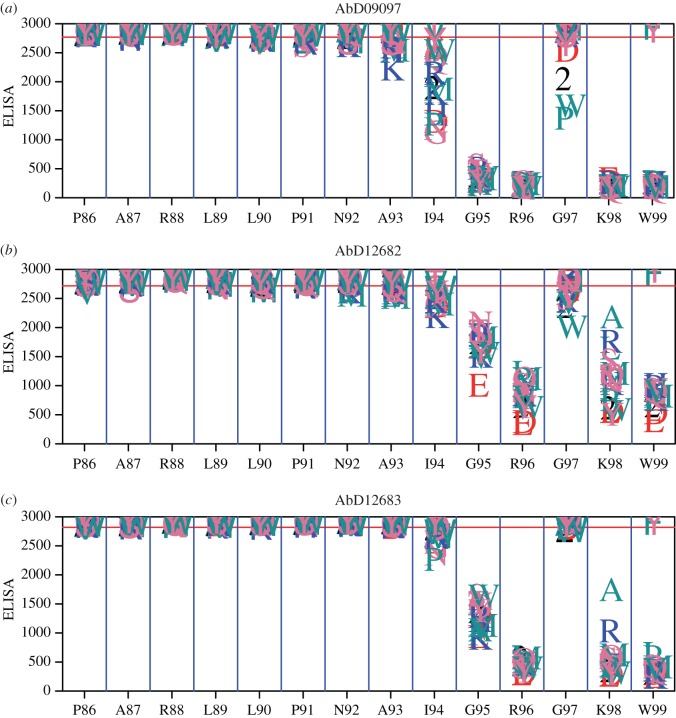
Peptide replacement array analysis for sclerostin-neutralizing Fabs on all single mutant variants of peptide PARLLPNAIGRGKW representing residues Pro86 to Trp99 of loop 2 of murine sclerostin. The red line represents the mean signal height that is recorded for the base peptide. At each position along the sequence, a letter representing the substitution is drawn at the height of the signal intensity recorded for that particular substitution. One letter amino acid code was used for all amino acids except Cys, for which the number 2 was used representing a cysteine protected with acetamidomethyl (ACM) to prevent formation of disulfide bonds. For all three Fabs, AbD09097 (*a*), AbD12682 (*b*) and AbD12683 (*c*), the first eight residues can be replaced by any other tested amino acid without altering binding of the peptide. Exchange of the succeeding six amino acids, representing residues Ile94 to Trp99 of sclerostin, similarly but not identically affect binding of the peptide by the different Fab proteins.

The epitope of AbD09097 was also analysed using peptide arrays. An array consisting of all overlapping 15mers of human and murine sclerostin readily identified the sequence GRVKWW (residues 95–100) to contribute most to binding. A slight preference for the murine sequence that contains valine at position 95 where the human form contains glycine was noted, but seems to be of minor importance. A first truncation analysis on all overlapping 4–24mer peptides of loop 2 derived from the human sequence corroborated the core epitope and revealed that optimal binding to peptides was achieved for constructs that terminated at Trp99. Peptides that included Trp100 or residues even more C-terminal were bound less avidly. In a third round we then refined the analysis to determine the optimal motif recognized by AbD09097. Hereby all residues in the two 14mer peptides PARLLPNAIGRGKW and NAIGRGKWWRPSGP were systematically and individually exchanged for all other proteinogenic amino acids except Ile. In addition a truncation series of these peptides was made to analyse the required length of the recognition motif (electronic supplementary material, figure S1). As can be seen from figure 5*a*, changes in Gly95, Arg96 and Lys98 are not tolerated at all and abrogate binding of AbD09097 (for an alternative representation of the data, see electronic supplementary material, figure S2). Trp99 can only be replaced by the aromatic amino acids Phe or Tyr. Exchanges of Ile94 and Gly97 affect binding to a lesser degree, but peptides containing Val at position 97 (as occurs in the murine sequence) are fully bound by AbD09097. These results are fully consistent with the NMR analysis of AbD09097. A large set of murine sclerostin variants was then used in an SPR analysis to study binding and recognition of the various Fabs in more detail (electronic supplementary material, table S2). From 16 available single mutants, mutations in three positions decreased binding affinity (Arg96, Trp99 and Arg101) to AbD09097. All three residues are also part of the region which has been exchanged in the variants Scl Δloop and Scl Alaloop, which both did not bind AbD09097 (see above). Furthermore, an intact finger 1 region seems also important, as binding of the AbD09097 to the variant Scl F1mut, which carries the mutations R56N, E57A, H59R, T61K, R62K, T65N, R70T and K73Q, was significantly lower, too. The five other antibodies selected against sclerostin were similarly tested by SPR. Consistent with our finding that not one of these variants is neutralizing sclerostin, none was affected by mutations in loop 2 (electronic supplementary material, table S2). Additional epitope mapping for some non-neutralizing Fabs using a similar peptide array as described above suggested that their epitope is either located in the N-terminal region (AbD09094, His42–Ala46) or in the finger 2-region (AbD09096: Gly126–Lys133, AbD09100: Gly125–Lys141, AbD09101: Gly125–Ala138, AbD09173: Gln117–Ala128). The fact that the only Fab recognizing an epitope in loop 2 of sclerostin shows good neutralization of Wnt signalling inhibition underlines the importance of the flexible loop of sclerostin for its bioactivity.

### 3.6. Crystal structure analysis of the neutralizing anti-sclerostin Fab AbD09097

**Figure 6.**
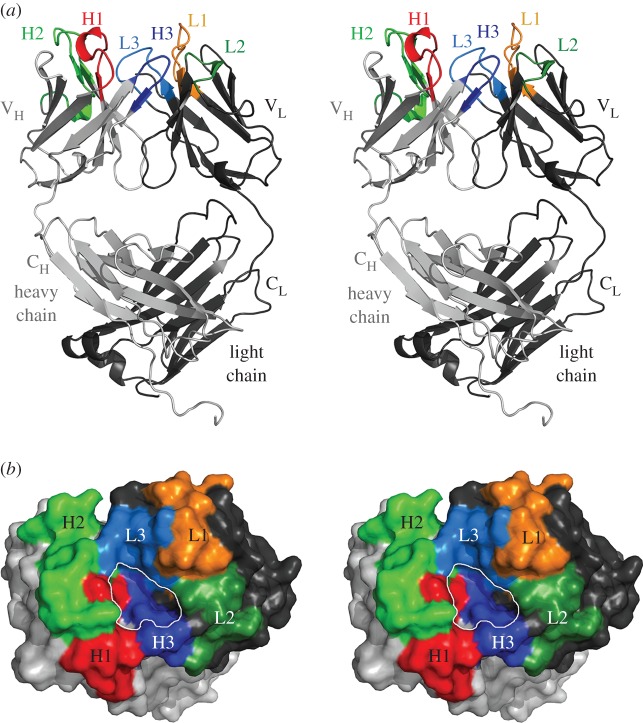
Crystal structure analysis of the sclerostin-neutralizing Fab AbD09097. (*a*) Stereo view of a ribbon plot of the Fab AbD09097. The constant (C) and variable (V) domains of the light (subscript L) and heavy (subscript H) chain are indicated. The six complementarity-determining regions (CDRs) are marked with L and H for the light and heavy chain. (*b*) Van der Waals surface representation (stereo view) of the antigen-binding site (as in (*a*) but viewed from the top). The CDR loops are colour-coded as in (*a*). A deep pocket (marked by a white line) is formed in the centre of the antigen-binding site, which is limited by the CDRs 1 and 3 of the light and heavy chain (L1, H1, L3 and H3). A small part of the CDR2 of the light chain (L2) and the heavy chain (H2) also contributes to the pocket.

To get insights into the paratope of a sclerostin-neutralizing antibody on a molecular level, we determined the structure of AbD09097 by X-ray crystallography at a resolution of 1.85 Å. The final model consists of residues Asp1 to Ala215 of the light chain and Gln1 to Gly224 of the heavy chain. In addition, 288 water molecules could be modelled and for five residues, i.e. Val37 and Glu150 of the heavy chain and Ser22, Lys104 and Arg143 of the light chain, alternative side chain conformations could be identified in the electron density map (figure 6*a*; for sequence see electronic supplementary material, figure S3). AbD09097 exhibits a classical Fab architecture with an elbow angle of 144°, the latter of which describes the orientation between the variable and constant domains by building pseudo-dyads between V_L_/V_H_ and C_L_/C_H_. For comparison, the BMPR1a-neutralizing Fab AbD1556 (PDB entry 3NH7 [52]), which also derives from phage display screening of the HuCAL GOLD library, has an elbow angle of 231°. Thus both Fabs can be superimposed only either on the variable region (RMSD for Cα positions: 1.25 Å) or the constant region (RMSD for Cα positions: 1.73 Å; electronic supplementary material, figure S4). This difference is probably due to the fact that the light chains of AbD09097 and AbD1556 only share 45% amino acid sequence identity as the AbD09097 light chain belongs to the kappa class, whereas that of AbD1556 is a member of the lambda subfamily (electronic supplementary material, figure S4). Conformingly, another Fab (e.g. a Fab directed against a K63-linked di-ubiquitin, PDB entry 3DVG [53]), which—like AbD09097—has a kappa light chain, resembles the same overall architecture as AbD09097 with a similar elbow angle of 165° (electronic supplementary material, figure S4).

The high resolution and the unambiguous electron density enabled a detailed analysis of the CDR loops forming the sclerostin-binding site. The six CDR loops (figure 6; electronic supplementary material, figure S3) harbour eight tyrosines and nine serines (among a total of 60 residues forming the antigen interaction site); two more tyrosine residues are located within the antigen-binding groove but are not part of the CDRs. The heaped occurrence of Ser and Tyr in the antigen-binding groove of AbD09097 is also found with other antibodies and might be explained with the involvement and importance of both amino acid types in hydrogen bond and/or hydrophobic interactions through either their π-electron system and/or hydroxyl group [54,55]. When using the Chothia nomenclature for CDR classification [56,57], the light chain CDR2 (length 7aa, Asp50 to Thr56, Kabat numbering is used throughout [58]) and CDR3 (9aa, Gln89 to Thr97) both belong to the class 1 (representative structures are PDBs 1LMK and 1TET, respectively) and the heavy chain CDR1 (10aa, Gly26 to His35) and CDR2 (17aa, Thr50 to Gly65) represent class 1 (similar to in PDB entry 2FBJ) and class 3-like (similar to in PDB 1IGC) canonical CDR loop, respectively (electronic supplementary material, figure S5). These classifications were confirmed by structural comparisons using reference Fabs (electronic supplementary material, figure S5). No grouping could be obtained for CDR1 of the light chain (length 12aa, Arg24 to Ala34) and CDR3 of the heavy chain (6aa, Trp95 to Ile102) if the classical rules of Chothia and co-workers were applied [56,57,59]. Newer antibody analysis tools like the AHo numbering scheme [60] and the North clustering [61] classify CDR1, 2 and 3 of the AbD09097 light chain to adopt loop architectures belonging to the L1-12-1, L2-8-1 and the L3-9-cis7-1 clusters, respectively. According to this analysis tool the three CDRs of the AbD09097 heavy chain resemble structures found in the H1-13-1 (CDR1), the H2-10-2 (CDR2) and the H3-8-1 (CDR3) clusters (electronic supplementary material, figure S5).

### 3.7. Crystal-lattice contacts provide hints for the sclerostin–Fab AbD09097 interaction

**Figure 7.**
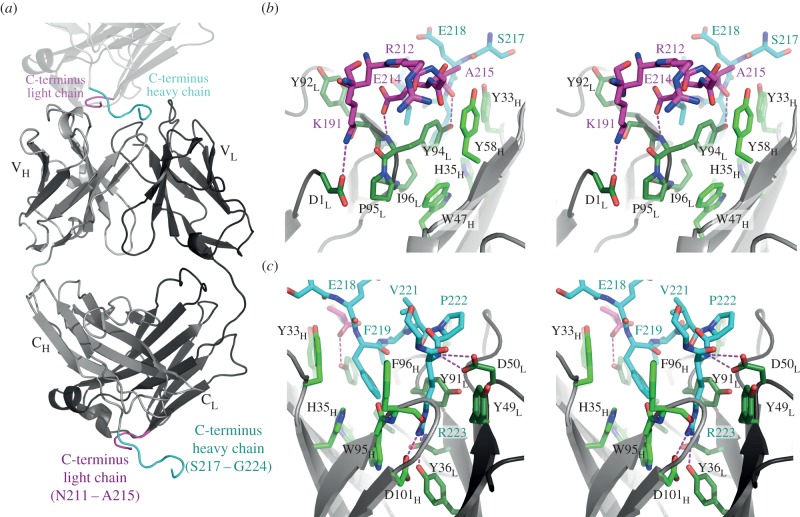
The C-termini of the Fab light and heavy chain potentially mimic the interaction of sclerostin with the Fab AbD09097. (*a*) Crystal-lattice contacts (the second Fab molecule is indicated in light grey, being stacked on top of the other Fab molecule) between symmetry-related Fab molecules result in the interaction of the C-terminus of the light chain (aa N211–A215 and K191) with the CDRs L1, L3, H2 and H3 of the antibody. Similarly, the C-terminus of the heavy chain comprising mainly the residues of the thrombin cleavage site for the removal of the Myc and hexahistidine-tag (aa S217–G224) interacts with the deep pocket formed by the CDRs L1, L2, L3, H1 and H3. (*b*) Magnification (stereo view) of the interaction of the C-terminus of the light chain (carbon atoms coloured in magenta) with the binding site of the Fab. Selected hydrogen bonds are marked as magenta stippled lines. Residues of the light chain are coloured in dark green and labels contain a subscript L. Residues of the heavy chain are coloured in green and labels contain a subscript H. Numbering of the residues located in the CDRs follows the rules of Kabat. (*c*) As in (*b*) but rotated around the *y*-axis by 180° to show the interaction of the C-terminus of the heavy chain (carbon atoms coloured in cyan) with the antigen-binding site of the Fab. Two amino acid residues of the thrombin cleavage site, F219 and R223, deeply penetrate into the pocket. Arginine 223 forms multiple hydrogen bonds emanating from its main and side chain atoms to residues Y36 and D50 of the light chain and F96 and D101 of the heavy chain.

The most prominent feature of the AbD09097 structure is seen when looking top down onto the CDR loops. The antigen-binding site is built by CDR2 and 3 of the light chain forming one side and the heavy chain CDRs 1 and 2 sculpting the other side, thereby creating a deep crevice or pocket (figure 6*b*). Two aromatic residues, Tyr94 of CDR3_L_ and Phe96 in the CDR3_H_, border the crevice. Tryptophan 95 of CDR3_H_ and Phe98 at the end of CDR3_L_ form the bottom of this pocket, which has a length of 12 Å, is about 7 Å wide and has a depth of 5 Å (figure 6*b*). Shape and size of this antigen-binding site of AbD09097 shares some similarities with that of other antibodies recognizing linear peptide motifs, e.g. Fab 8F5 that binds the rhinovirus capsid protein VP2 (PDB entry 1A3R, [62]), or Fab fragment 59.1, which interacts with the third variable loop (V3) of the HIV-1 protein GP120 (PDB entry 1ACY [63]). Our mapping studies indeed indicated that AbD09097 recognizes a linear peptide, which comprises residues Leu90 to Asp106 according to NMR chemical shift mapping (figure 4) and with the peptide array replacement analysis suggesting Ile94 to Trp99 as the main binding determinants of this peptide motif (figure 5). To our surprise a detailed analysis of the crystal symmetry then revealed that the crevice-like antigen-binding site of AbD09097 is already filled with two peptides originating from a symmetry-related AbD09097 molecule in the crystal lattice (figure 7; electronic supplementary material, figure S3*c*,*d*).

Most importantly, these two peptides lining the pocket potentially mimic some of the sclerostin loop 2–Fab interactions. One peptide originates from the C-terminus of the light chain and consists of residues Asn211 to Ala215 (figure 7*b*; electronic supplementary material, figure S3). It shares various van der Waals contacts with CDR3_L_ and CDR2_H_ and has a buried surface area of about 140 Å^2^. It, furthermore, forms two hydrogen bonds, one between the carboxylate group of Glu214 and the amide of Tyr94 (CDR3_L_) and one between the C-terminal carboxylate group of Ala215 and the hydroxyl group of Tyr94 (CDR3_L_) (figure 7*b*). The interaction of this peptide segment with the antigen-binding site is complemented by Lys191 from the light chain of the same symmetry-related Fab, which engages in a further hydrogen bond between its side chain amine group and the N-terminal Asp1 of the light chain (figure 7*b*).

The second peptide occupying the antigen-binding site is part of the C-terminus of the AbD09097 heavy chain. It carries a 29aa extension harbouring a thrombin cleavage motif, a myc- and a hexahistidine-tag for detection and purification purposes (electronic supplementary material, figure S3). It is important to note that this peptide tag could not be cleaved off without destroying the high-resolution diffraction properties of the Fab crystals, highlighting the importance of these interactions for proper crystal-lattice formation (see also [49]). On the other hand, gel-filtration of AbD09097 did not provide hints for oligomer formation in solution suggesting that the homomeric interactions of AbD09097 with C-terminal peptide sequences are weak. The low affinity of this interaction is probably due to the limited similarity of the C-terminal tag-derived peptide sequence compared with the ‘natural’ target sequence in the sclerostin loop 2. Of this crevice-occupying peptide, residues Ser217 to Phe219 originate from the Fab heavy chain, and residues Leu220 to Gly224 are already part of the recognition sequence for thrombin. This peptide snugly fits into the deep pocket in the Fab antigen-binding site burying about 470 Å^2^ surface area (figure 7*c*). Whereas Ser217 and Glu218 make few van der Waals contacts with CDR1_H_, Phe219 deeply penetrates into a hydrophobic pocket formed by Tyr33 and His35 (CDR1_H_), Tyr91, Tyr94, and Ile96 (CDR3_L_) and Trp95 (CDR3_H_). Leu220, the first residue of the thrombin cleavage motif, also engages in hydrophobic interactions with Tyr94 and Ile96 of CDR3_L_. The succeeding Val221 and Pro222 bulge out of the crevice and thus share only little contact with the CDR1_L_ of AbD09097. By contrast, the side chain of Arg223 deeply immerses into the pocket with its guanidinium group forming a tri-dentate hydrogen bond with the carbonyl of Phe96, the carboxylate group of Asp101 of CDR3_H_ and the hydroxyl group of Tyr36 C-terminal of CDR1_L_. In addition, the backbone amide of the arginine residue is fixed to Asp50 of CDR2_L_ by two hydrogen bonds. The multiple hydrogen bonds and the large surface area buried upon complex formation suggest that the arginine and the preceding phenylalanine are key binding elements for motifs recognized by AbD09097 and they might be also present in a similar arrangement in loop 2 of sclerostin recognized by the neutralizing Fab. Our peptide array replacement study indeed indicated that a rather short peptide comprising residues Ile94 to Trp99 of sclerostin harbours the key motif for binding to AbD09097 (figure 5). Together with the notion that AbD09097 binds murine sclerostin with higher affinity than the human protein and the fact that the only difference within the motif IGRVKW is a Gly-to-Val exchange, this suggests that a potential minimal sequence motif for binding of sclerostin to AbD09097 is (hydrophobic-G)-R-hydrophobic-K-aromatic. This also probably explains how AbD09097 neutralizes sclerostin activity, as sclerostin's key determinant for binding to the Wnt co-receptor LRP5/6 is the NXI-motif, which just precedes the antibody recognition motif. Thus binding of sclerostin to the antibody competes with its capability to bind to LRP5/6 and, therefore, the neutralizing antibody releases LRP5/6 from a complex with sclerostin thereby rescuing Wnt signalling.

### 3.8. Affinity maturation of the sclerostin-neutralizing antibody AbD09097

AbD09097 efficiently neutralized sclerostin activity in different cellular assays [36,64], but was more effective on murine than on human sclerostin. This correlates with the more than fivefold higher binding affinity of AbD09097 for the murine isoform. Fusing two AbD09097 Fabs into the bivalent Fab AbD12533 (dimerized using a helix-turn-helix motif, [65]) strongly increased the binding affinity also for the human isoform in our *in vitro* SPR analyses (table 2). However, this increase is likely to be biased by artificial avidity effects, which are due to the specific SPR set-up using sclerostin as immobilized ligand and perfusing the antibody proteins as analytes.

**Table 2.** Affinity maturation of the Fab AbD09097. Analysis of Fab antibodies derived from affinity maturation of the ancestor Fab AbD09097. The Fab AbD12533 is a bivalent AbD09097, in which two AbD09097 were fused by a helix-turn-helix motif [65]. Fab specificity was screened using an ELISA employing a panel of control proteins, and murine and human sclerostin. The numbers represent signal/noise ratios (fold background). The binding affinity of these Fabs were determined using surface plasmon resonance (SPR), *K*_D_ values and kinetic parameters were obtained using a set-up with the Fab antibody proteins as analytes (six different concentrations) and human and murine sclerostin as immobilized ligand. Fold change represents the affinity enhancement compared with the binding affinity of the ancestor Fab AbD09097. Species-specificity was calculated from 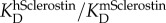.

AbD	ELISA					SPR								species-specificity
	controls			sclerostin		murine sclerostin				human sclerostin				
	BSA	CD33	GST	mScl.	hScl.	*k*_on_ (×10^3^ M^−1^ s^−1^)	*k*_off_ (×10^−4^ s^−1^)	*K*_D_ (nM)	fold change	*k*_on_ (×10^3^ M^−1^ s^−1^)	*k*_off_ (×10^−4^ s^−1^)	*K*_D_ (nM)	fold change	
AbD09097	1	1	1	39	34	4.4	1.8	46	—	6.6	16	260	—	5.7
AbD12533						10	0.09	10	4.6	130	2.2	18	14	1.8
AbD12681	1	1	2	26	44	17	5.4	35	1.3	27.4	16	66	3.9	1.9
AbD12682	1	1	5	44	57	22	2.5	13	3.5	32	6.6	23	11	1.8
AbD12683	1	1	1	28	64	3.4	<0.01	<1	>40	6.4	3.3	53	4.9	>53
AbD12684	1	1	4	53	73	18.4	2.5	15	3.1	25.3	7.3	32	8.1	2.1
AbD12685	1	1	5	46	61	13	2.9	145	0.3	20.4	10	56	4.6	0.4
AbD12686	1	1	1	55	84	5.6	0.14	3	15	9.5	5.9	66	3.9	22
AbD12687	1	1	1	50	57	16	17	120	0.4	23.3	47	228	1.1	1.9

Therefore, to obtain a neutralizing antibody with improved affinity for human sclerostin we applied affinity maturation to AbD09097 employing an alternative panning strategy. As the heavy and the light chain CDR3 loops form the most inner part of the antigen-binding groove (figure 6*b*), they are most important for binding affinity and are thus the prime target for affinity maturation approaches. The HuCAL GOLD phage library was generated with all six CDRs being diversified to ensure a large antibody repertoire, but special emphasis was directed on diversification of the heavy chain CDR3. This suggests that HuCAL GOLD-derived antibodies have rather ‘optimal’ CDR3_H_ sequences. For affinity maturation, we thus targeted CDR3_L_ by replacing the original CDR3_L_ with a highly diverse cassette to select Fabs with CDR3_H_ of the ancestor AbD09097 but having newly selected loops for CDR3_L_. This targeted library was then subjected to a solution panning using decreasing amounts of biotinylated human sclerostin and more stringent washing to ensure selection of high-affinity binders. From more than 100 hits obtained in ELISA, the 20 clones with the highest signal were sequenced and seven unique new antibodies (AbD12681–AbD12687) were obtained. In ELISA, all seven Fabs bound both human and murine sclerostin with a good signal (table 2). SPR analysis showed that for five Fabs the affinity to human sclerostin was improved fivefold or better (table 2). In contrast with the significant affinity enhancement for human sclerostin, binding to murine sclerostin either improved only marginally, with three Fabs (e.g. AbD12681, 12682 and 12684) showing an increase in affinity of threefold or less and two Fabs, AbD12685 and 12687, even binding less tightly to murine sclerostin when compared with their ancestor AbD09097 (table 2). Only two Fabs, AbD12683 and 12686, bound murine sclerostin with strongly increased affinities (46- and 15-fold, table 2), indicating that optimizing CDR3_L_ sequences using human sclerostin during selection can also benefit binding and recognition of the murine isoform. Most importantly, of the seven new Fabs, three antibodies (AbD12681, 12682, 12684) exhibit not only improved affinities for human sclerostin (4- to 11-fold) but also have a strongly reduced preference (calculated from 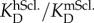) for murine sclerostin, which decreased from sixfold of the ancestor AbD09097 to twofold or less (table 2). Thus, these three antibodies bind murine and human sclerostin in a non-discriminatory manner and might, therefore, neutralize both sclerostin isoforms with similar efficacy. With AbD12685 a Fab was obtained that binds murine sclerostin with lower affinity than its ancestor AbD09097 and at the same time exhibits an increased affinity for human sclerostin. Thus, this Fab binds preferentially the human isoform. From this data, it seems that the set of affinity-maturated Fabs AbD12681–AbD12687 can be grouped into two classes: one group (group 1) consisting of AbD12681, 12682, 12684 and 12685, binds both isoforms, murine and human sclerostin, in a non-discriminatory manner or even with preference for the human isoform; the second class (group 2) comprises AbD12683 and 12686 and still strongly favours murine over human sclerostin like the ancestor Fab AbD09097.

To get insights into how the changes in CDR3_L_ altered recognition of the sclerostin epitope, we first performed SPR analyses using our set of sclerostin variants (electronic supplementary material, table S2). The data show that recognition of the key determinants Arg96 and Trp99 is preserved in all affinity-maturated Fabs. Mutation of other residues in loop 2 seems not to influence binding of the affinity-maturated Fabs differently compared with AbD09097, except for Arg101, which was required for high-affinity binding to AbD09097 but is dispensable for binding of the AbD1268X series (electronic supplementary material, table S2). Only mutations in finger 1 (i.e. Scl F1mut) seem to affect a subset but not all affinity-maturated Fabs (AbD12681, 12682, 12684). Given the rather large changes in binding affinity the SPR analysis did not, however, provide a conclusive picture how the affinity maturation affects recognition of the sclerostin loop 2 epitope. Thus, we applied a peptide replacement and truncation array analysis for the Fabs AbD12682, representing the group 1 Fabs, and AbD12683, as a member of group 2 Fabs (figure 5; electronic supplementary material, figure S1). Here peptide variant mimicking residues Pro85 to Trp99 of sclerostin were prepared with each residue of the peptide replaced by all other proteinogenic amino acids except Ile. The results suggest that the core epitope recognized by affinity-maturated Fabs is possibly smaller as mutation of Ile94 affects binding less than AbD09097. Similarly Gly95, which was insignificant for high-affinity binding to AbD09097, can be replaced by other amino acid types without fully abrogating binding. Two positions, however, support the above classification for the affinity-maturated Fabs into two classes: with AbD09097 and AbD12683, replacement of the Arg96-equivalent residue in the peptide is detrimental to binding, whereas AbD12682 seem to tolerate exchange to non-acidic amino acids without completely losing binding affinity. Lys98 was also essential for binding to AbD09097; with AbD12683 exchange of this lysine for amino acids other than alanine leads to a complete loss of binding. With AbD12682, on the other hand, many amino acid types seem to decrease binding only to a minor degree. The hypothesis that AbD12683/group 2 Fabs and AbD09097 share a similar recognition mechanism while the binding of group 1 Fabs (as represented by AbD12682) diverged further from the ancestor AbD09097 is even more evident from the peptide truncation array analysis (electronic supplementary material, figure S1). Here, the binding of AbD09097 and AbD12683 to the loop 2 mimicking peptide is almost completely lost upon any C-terminal truncation of the 14mer peptide, indicating the importance of Trp99 in the recognition motif. By contrast, both Fabs tolerate removal of the N-terminal residues up to Ala93. AbD12682 instead does still bind peptides in which Gly97 to Trp99 were removed with moderate affinity, albeit high affinity binding requires peptides with similar sequence and length to AbD09097 and AbD12683.

**Figure 8.**
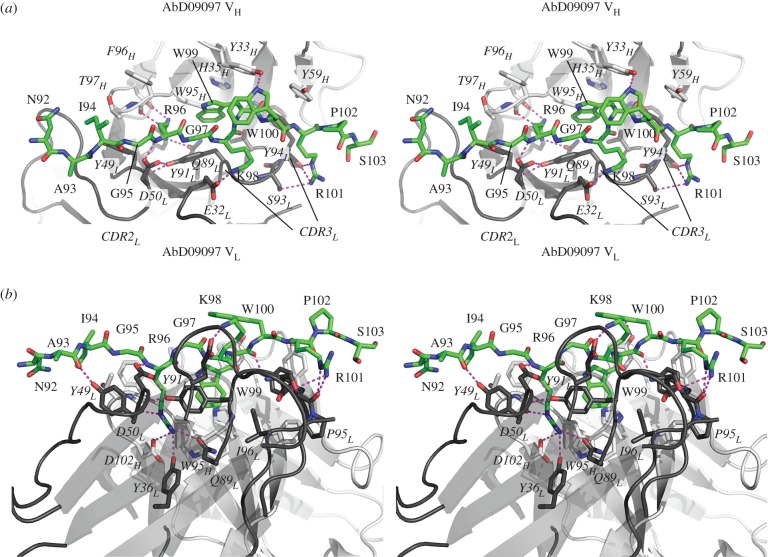
Model of the sclerostin–AbD09097 complex. On the basis of the C-termini of the heavy and light chains of a symmetry-related Fab molecule in the crystal lattice of the AbD09097 crystal a theoretical model was built to show the loop 2 of human sclerostin comprising Asn92 to Ser103 bound into the antigen-binding crevice of AbD09097. The carbon atoms of the heavy and light chains of AbD09097 are coloured in light and dark grey, respectively. The carbon atoms of the sclerostin loop are shown in green. Potential hydrogen bonds between the sclerostin loop and Fab are indicated by stippled lines in magenta. (*a*) Top down view of the Fab-binding cleft. (*b*) As in (*a*) but rotated 90° clockwise around the *x*-axis.

With the data of the mapping study of the affinity-maturated Fabs and the crystal structure of AbD09097 with two sclerostin-mimicking peptides in the antigen-binding site at hand (figure 7*b*,*c*), we could build a molecular model showing how sclerostin potentially binds to the neutralizing Fabs. Here loop 2 binds along the crevice of Fab AbD09097 with Arg96 occupying the same deep cleft formed by CDR3_H_ and CDR2_L_ and engaging in similar hydrogen bonds with Asp101_CDR3H_ and Tyr36_CDR2 L_ as the Arg223 residue in the C-terminus of the symmetry-related Fab moiety (figure 8). Trp99 is also buried inside the cleft forming hydrophobic interactions with Tyr33 and His35 of CDR1_H_ and Tyr91 of CDR3_L_ similar to the symmetry-related Phe219 as seen in the crystal structure (figure 8). The main difference between the peptide in the crystal structure and the peptide in the sclerostin–AbD09097 complex model is that the direction of the peptide backbone had to be reversed. In the current model, Lys98 and Trp100 of the loop 2 are both located outside the antigen-binding crevice, with Lys98 potentially interacting with Glu32 of CDR1_L_ via a saltbridge. The different impact of the R101A mutation on binding seen for the ancestor AbD09097 and the affinity-maturated Fabs strongly suggests that Arg101 is placed on top of CDR3_L_ where it might interact with Asp1 of the light chain. Owing to the different length and amino acid composition of CDR3_L_ this interaction might be disrupted in the affinity-maturated Fabs thereby explaining why Arg101 is not required for high-affinity binding to these Fabs (electronic supplementary material, figure S6). However, for a detailed structure–function analysis an experimental structure of sclerostin or a sclerostin-derived peptide bound to AbD09097 is needed in the future.

## 4. Discussion

New therapy concepts for bone-eroding diseases such as osteoporosis attempt to restore bone density rather than only impeding further resorption, which is the mechanism of action for classical osteoporosis drugs such as bisphosphonates [66]. Two new key targets that may allow for a bone restoration have been identified, the tumour necrosis factor (TNF) ligand RANKL and the Wnt antagonist sclerostin [67]. RANKL is associated with osteoclastogenesis and its expression can lead to bone loss (for review, see [68]). Conformingly, a neutralizing anti-RANKL antibody (denosumab) showed bone-restoring properties and was approved for treatment of osteoporosis in mid-2010 [69]. Sclerostin was initially discovered from diseases with a bone-overgrowth phenotype, sclerosteosis and the van Buchem disease [8,70]. Here lack of sclerostin leads to osteopetrosis indicating that it is a negative regulator of bone growth. Various studies have shown that it inhibits the Wnt/β-catenin signalling pathway [11,12,71,72]. By binding to the Wnt co-receptor LRP5/6 ectodomain, it competes with Wnt ligands, which signal via complex formation with LRP5/6 and receptors of the Frizzled family. Structure–function studies identified loop 2 of sclerostin as the key binding determinant for LRP5/6 [36,37,41]. Therefore, antibodies targeting the LRP5/6 binding motif in the second loop of sclerostin should potentially be neutralizing with respect to sclerostin-mediated Wnt inhibition.

For functional studies, we generated Fab antibodies against sclerostin and investigated their properties using biochemical and cell-based assays. From the 11 Fab antibodies initially obtained, a reportergene-based cellular assay with Wnt1 as stimulant identified one Fab (AbD09097) to effectively neutralize sclerostin thereby rescuing sclerostin-attenuated Wnt1 activity [64]. Epitope mapping revealed that AbD09097 recognizes loop 2 of sclerostin and blocks access to the NXI-motif, the latter of which is required for binding to LRP6 [36,37,41]. Thus, neutralization by AbD09097 seems simply due to competing off an antagonist (sclerostin), which competes with Wnt factors for binding to a receptor (LRP5/6). Consistent with the above mechanism, Fabs that bound to either N- or C-terminus or within finger 1 and/or 2 did not neutralize sclerostin-mediated Wnt1 inhibition.

Analysis of crystal-lattice contacts in the crystal structure of AbD09097 provided first hints into how this Fab recognizes and binds loop 2 of sclerostin and how this interaction blocks sclerostin from binding to the Wnt co-receptor LRP6. A short segment at the C-terminus of the Fab heavy chain, which is part of a thrombin protease recognition site (-FLVPR↓GS-) and which was required to obtain X-ray diffracting crystals [49], filled the antigen-binding crevice of the Fab potentially mimicking sclerostin-AbD09097 key interactions. Namely, the phenylalanine and arginine residue of the above sequence engage in intimate contacts with the antigen-binding cleft. The phenylalanine shares hydrophobic and ππ-stacking interactions with aromatic residues in CDR1_H_ and CDR3_L_, and the arginine forms multiple hydrogen bonds with a buried Asp and Tyr residue in CDR3_H_ and CDR2_L_, respectively. Despite the high resolution of 1.85 Å, which allowed identification of more than 280 water molecules around the protein, no water was observed inside the peptide–Fab interface and this lack of polar solvent molecules probably strengthens the intermolecular polar and hydrophobic interactions. The apparent similarity of this peptide with the sclerostin epitope for AbD09097 as derived from our various mapping studies allowed building a model for the sclerostin–AbD09097 interaction. Data from affinity maturation of AbD09097 helped to orient the loop 2 of sclerostin within the antigen crevice. As only CDR3_L_ changed during maturation and the contribution of Arg101 to binding was markedly altered during this process, Arg101 is very likely located at or close to CDR3_L_ thus requiring Arg96 and Trp99, the two other residues essential for binding to AbD09097, to be located in the deep cleft formed by CDR1 and 3 of the heavy chain and CDR2 of the light chain (figure 8).

With this model and the sequences of CDR3_L_ (electronic supplementary material, figure S6*a*) of the newly selected Fabs, we are able to propose molecular mechanisms explaining affinity maturation of AbD09097. The CDR3_L_ loop of the ancestor AbD09097 has the sequence QQYYSYPI, of which only the first Gln, the first Tyr and the sequence YPI face the inside of the peptide-binding crevice, suggesting that exchange of the other loop residues might not alter sclerostin binding. However, while the second glutamine does not face the peptide, it very likely is, nevertheless, essential for sclerostin binding, as it imprints and stabilizes the backbone conformation of CDR3_L_ via four hydrogen bonds. In a similar manner the first tyrosine of this 8mer sequence also engages in hydrogen bonds with residues of CDR2_L_ thereby forming a defined loop structure in CDR2_L_. The C-terminal sequence YPI lines the ‘end’ of the peptide-binding crevice representing a hydrophobic levee at the end of the deep cleft. To adopt the required turn conformation the proline residue is configured with a *cis*-peptide bond. When now comparing the alternative CDR3_L_ sequences of the seven affinity-maturated AbD09097 descendants, all seven sequences, therefore, share the double glutamine sequence, six out of seven have the proline–isoleucine motif and five also carry either a tyrosine or a phenylalanine residue ahead of the -PI- motif. Modelling of AbD12681, 12682 and 12684 suggests that all three Fabs have an identical peptide-binding crevice sharing a consensus motif -QQDXXFPI- in which the residues X are either a histidine, glutamate, valine or serine (electronic supplementary material, figure S6*a*,*b*). This similarity is reflected in rather similar binding properties. All three Fabs do not discriminate between the murine and human isoform (i.e. the affinity difference is 2 or less) and bind murine and human sclerostin with affinities of about 15–35 nM and 25–65 nM (table 2). The commonalities of all three Fabs are the exchange of the first tyrosine against an aspartate and the replacement of the tyrosine of the YPI-motif with a phenylalanine. Whereas the latter exchange slightly enlarges the hydrophobic pocket to possibly better accommodate Trp99 (figure 8), exchange of tyrosine against aspartate firstly provides CDR2_L_ with increased flexibility due to a loss of the fixating hydrogen bonds emanating from the tyrosine hydroxyl group and secondly adds an additional negative charge at the bottom of the deep cleft, which very likely houses Arg96 (figure 8; electronic supplementary material, figure S6*b*). In this context, it is important to note that the affinity enhancement of these three Fabs is rather due to an increased association rate (approx. four- to fivefold), whereas the slowdown in dissociation, usually the driver in affinity maturation, is only approximately two- to threefold. The binding of sclerostin to the ancestor Fab AbD09097 is characterized by a quite slow association (*k*_on_ 4 × 10^3^ M^−1^ s^−1^) and a slow dissociation (*k*_off_ 1 × 10^−4^ s^−1^) indicating that affinity is limited more by the rate of complex formation rather than the complex stability. Thus, widening of the binding cleft and placement of an additional negative charge to foster the fixation of Arg96 in the deep pocket might very well speed up the interaction thereby enhancing affinity by facilitating complex formation instead of contributing to complex stability. The non-discriminatory binding to murine and human sclerostin found for these three Fabs might also be associated with the Tyr-to-Asp mutation in the CDR3_L_. The only difference in the Fab-binding motif in loop 2 between murine and human sclerostin is the exchange of Gly97 for valine. The removal of the conformational fixation of CDR3_L_ by the Tyr-to-Asp exchange (see above) provides more flexibility to the binding pocket to better accommodate the human loop peptide segment (figure 8; electronic supplementary material, figure S6). The latter is more rigid as the valine residue cannot adopt backbone conformations amenable to glycine. Consistent with this hypothesis, the two affinity-maturated Fabs that exhibit an even higher preference for murine sclerostin, AbD12683 and 12686, both carry a tyrosine at position 3 of CDR3_L_. The molecular reason why both Fabs have, nevertheless, a 15- to 40-fold higher affinity than the ancestor AbD09097 although the N-terminal loop sequence is unaltered might be in the change of the YPI-motif at the C-terminus end of CDR3_L_. But as both Fabs have rather different amino acid sequences, experimental structures of either one of both Fabs or AbD09097 in complex with a loop 2-derived peptide are necessary to understand the details of sclerostin recognition by this set of neutralizing Fabs.

## 5. Conclusion

In summary, the Fab antibodies obtained and characterized in this study provide a new and valuable tool set for studying the molecular mechanism of sclerostin. Among those, the sclerostin-neutralizing Fab AbD09097 (available as a full-length IgG as HCA230Z from AbD Serotec) or its affinity-maturated descendants might present the most interesting antibodies. Their high efficiency to block sclerostin might facilitate the development of new drugs targeting diseases characterized by bone loss such as osteoporosis.

## Supplementary Material

Supplementary Material

## Data accessibility

The coordinates and structure factors have been submitted to the PDB RCSB databank (http://www.rcsb.org) using the accessible code 5DR5. The amino acid sequences of the neutralizing Fab AbD09097 and the affinity-maturated variants thereof are provided in the electronic supplementary material. All antibodies in this study are available from AbD Serotec.
