# Supplementary material for: The sclerostin-neutralizing antibody AbD09097 recognizes an epitope adjacent to sclerostin's binding site for the Wnt co-receptor LRP6
Source: Open Biol. 2016 Aug 24;6(8):160120. doi: 10.1098/rsob.160120 (PMC5008011; doi:10.1098/rsob.160120)
Supplement: Supplementary Material [file rsob160120supp1.pdf]

Supplementary table 1: Data acquisition and structure refinement

Crystal and dataset	AbD09097
Beamline	Rigaku MicroMax007 HF, detector Rigaku R-AXIS HTC
Wavelength	1.5418 Å
Space group	$P2_1$
Unit cell	a = 45.19Å; b = 78.50Å; c = 59.20Å<br>α, γ = 90°, β = 95.7°
Resolution range[a]	20.8 – 1.85 Å<br>(1.92 – 1.85)
number of reflections collected	126045 (8229)
number of unique reflections	34408 (2600)
Completeness	97.9% (80.1%)
Redundancy	3.7 (2.9)
R_{sym}[b]	0.068 (0.300)
$\langle I/\sigma(I)\rangle$	10.5 (3.7)
χ^2	0.97 (1.07)
**refinement statistics**	
R_{cryst}[c]	18.8 (36.1) %
R_{free} (5% test set)	24.4 (40.2) %
r.m.s. deviation[d]	
bonds	0.018 Å
angles	2.006°
torsion period 1	7.563°
torsion period 2	36.145°
torsion period 3	16.049°
torsion period 4	23.529°
average B-factor	22.5 $Å^2$
coordinate error (based on R_{free})	0.15 Å
TLS groups	4 (C_H, C_L, V_H, V_L)
Rampage analysis[e]	
residues in favored region	95.4% (415)
residues in allowed region	4.6% (20)
residues in outlier region	0.0% (0)

[a] values for the highest resolution shell are provided in parentheses

[b] $R_{sym} = \frac{\sum_{hkl}\sum_{i=1}^{n}|I_i(hkl) - I_i(\overline{hkl})|}{\sum_{hkl}\sum_{i=1}^{n} I_i(hkl)}$

[c] $R_{cryst} = \frac{\sum||F_{obs}| - |F_{calc}||}{\sum|F_{obs}|}$

[d] calculated with refmac version 5.8.0037

[e] Ramachandran/backbone torsion angle analysis

	ΔLoop	Alaloop	F1 mut	F2mut	R56A	R62A	R70A	P86D	R88A	L90A	P91D	N92A	I94A	R96A	W99A	W100A	R101A	N103A	R113A	R118A	R130A
**AbD09094**	+	+	+	+	+	+	+	+	+	+	+	+	+	+	+	+	+	+	+	+	+
**AbD09095**	+	+	-	nd	+	+	+	+	+	+	+	+	+	+	+	+	+	+	+	+	+
**AbD09096**	+	+	+	nd	+	+	+	+	+	+	+	+	+	+	+	+	+	+	+	+	-
**AbD09097**	-	-	-	-	+	nd	nd	+	+	+	+	+	+	-	-	+	-	+	nd	nd	nd
**AbD09098**	+	+	+	nd	+	+	+	+	+	+	+	+	+	+	+	+	+	+	+	+	+
**AbD09099**	+	+	+	nd	+	+	+	+	+	+	+	+	+	+	+	+	+	+	+	+	+
**AbD12681**	-	-	+	nd	+	nd	nd	+	+	+	+	+	+	-	-	+	+	+	nd	nd	nd
**AbD12682**	-	-	+	nd	+	nd	nd	+	+	+	+	+	+	-	-	+	+	+	nd	nd	nd
**AbD12683**	-	-	nd	nd	nd	nd	nd	+	+	+	+	+	+	-	-	+	+	+	nd	nd	nd
**AbD12684**	-	-	+	nd	+	nd	nd	+	+	+	+	+	+	-	-	+	+	+	nd	nd	nd
**AbD12685**	-	-	-	nd	+	nd	nd	+	+	+	+	+	+	-	-	+	+	+	nd	nd	nd
**AbD12686**	-	-	-	nd	+	nd	nd	+	+	+	+	+	+	-	-	+	+	+	nd	nd	nd
**AbD12687**	-	-	-	nd	+	nd	nd	+	+	+	+	+	+	-	-	+	+	+	nd	nd	nd

Supplementary table 2: SPR analysis of the Fab antibodies selected initially against murine sclerostin (AbD090..) and the affinity-maturated Fabs (AbD126..) against a set of single- and multi-mutation variants of murine sclerostin to determine the binding determinants for the sclerostin-Fab interaction. + marks that binding is not significantly altered by the mutation compared to the interaction between wildtype murine sclerostin and the respective Fab. - indicates variants, which show a markedly decreased binding affinity (≥ 5fold) for the respective Fab compared to wildtype sclerostin; nd not determined. The sclerostin multi-variants Δloop, Alaloop, F1mut and F2mut are described in the main text.

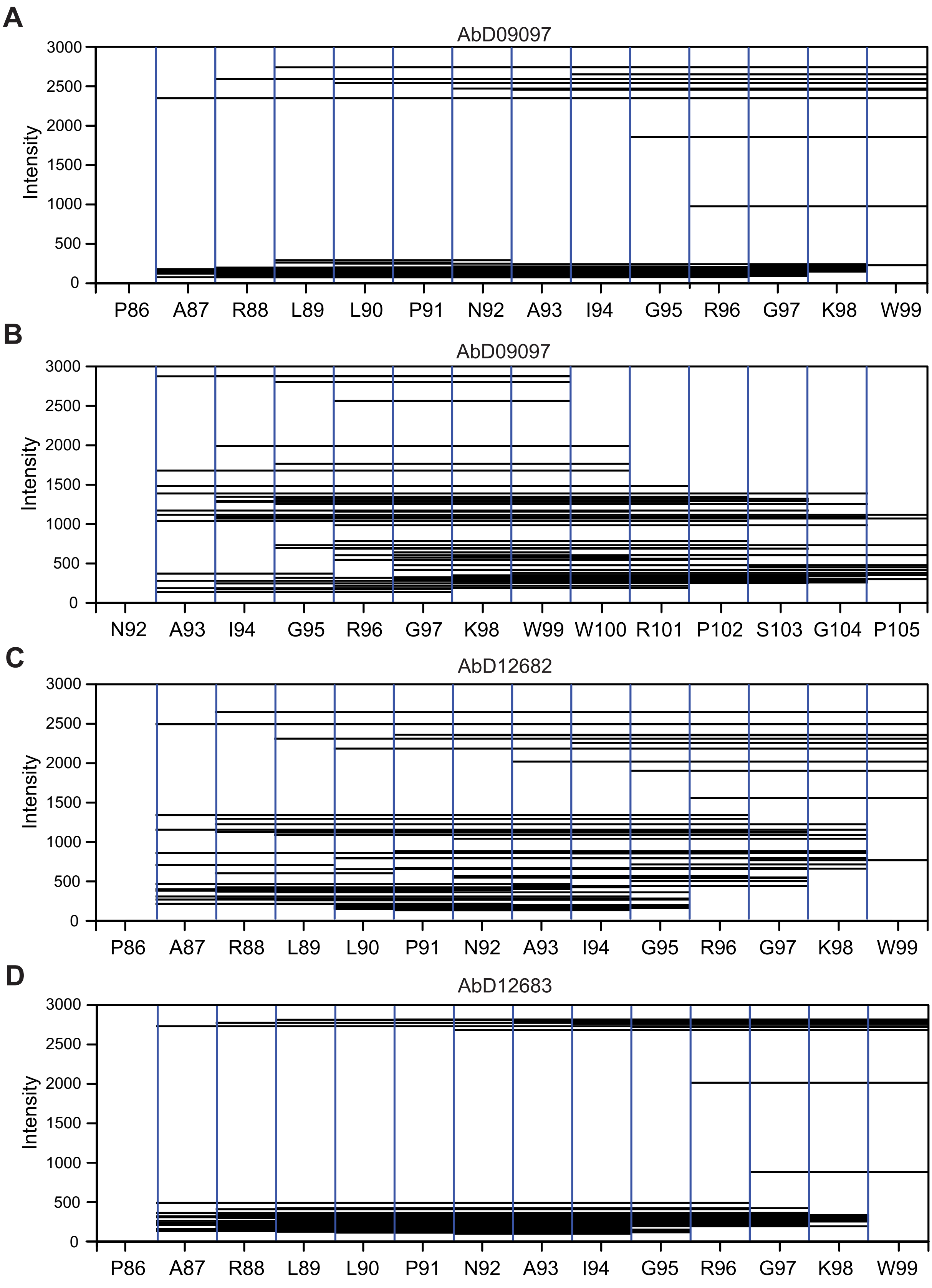

Supplementary figure 1: Peptide truncation array analysis of a 14mer peptide representing the loop 2 of murine sclerostin. For the analysis of the core epitope of the sclerostin-neutralizing Fab AbD09097 two peptides were used in the array, one equivalent to residues Pro86 to Trp99 (A) and a second peptide equivalent to residues Asn92 to Pro105 (B). The truncation analysis for the first analysis shows that removal of C-terminal residues starting with Trp99 leads to a strong reduction of

Fab binding, while removal of the first eight amino acids up to Ala93 is tolerated and does not seem to affect binding affinity significantly. Using the second peptide reveals that AbD09097 preferentially binds peptides ending at the first tryptophan residue (equivalent to Trp99) as truncated peptides ending at Trp99 bind with a higher signal than peptides extending past Trp99. A similar truncation array analysis was also performed for the two Fabs AbD12682 (C) and AbD12683 (D) each representing one of the two classes of seven affinity-maturated Fabs. In contrast to AbD12683, which seems to exhibit binding properties similar to the ancestor AbD09097, the Fab AbD12682 does tolerate the removal of Trp99 without completely loosing binding to the peptide if the peptide is sufficiently long and starts with the N-terminal residues Ala87 or Arg88. When comparing the pattern of the truncation analysis for the ancestor AbD09097 and the two affinity-maturated Fabs AbD12682 and AbD12683, the similarity in the binding preference clearly shows that AbD09097 and AbD12683 use a similar sclerostin epitope, whereas the epitope of AbD12682 diverged during affinity maturation.

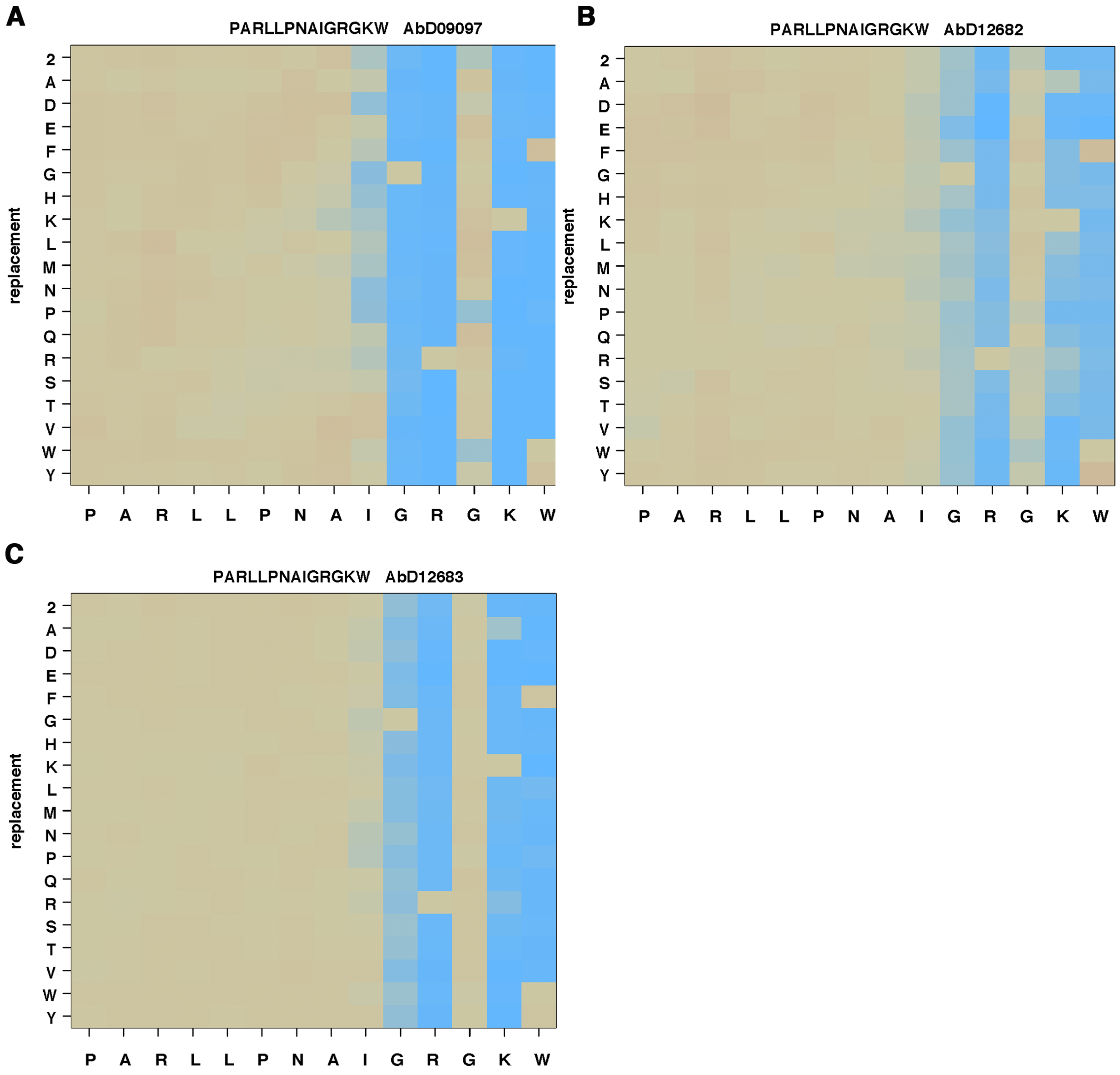

Supplementary figure 2: Peptide array replacement analysis as shown in figure 5 (main text), however the data are presented in a different manner. The x-axis indicates the amino acid residue as in the wildtype peptide, the y-axis lists the amino acid replacement (all proteinogenic amino acids except Ile). Thus each square in this two-dimensional plot represents the binding (as determined by ELISA) of one peptide (each plot shows a total of 266 peptides) in which the amino acid indicated on the x-axis was replaced by the amino acid as listed on the y-axis. The binding strength of each of these peptides to the respective Fab antibody is color-coded with bluish to blue colored squares indicating that binding is weaker compared to the wildtype peptide, beige representing binding that is comparable to the wildtype peptide and reddish to red colors marking variants that exhibit increased affinity for the Fab protein when compared with the wildtype peptide.

A

DIVLTQSPATLSLSPGERATLSCRASQSISSNELAWYQQ
KPGQAPRLLIYDTSNRATGVPARFSGSGSGTDFTLTISSL
EPEDFAVYYCQQYYSYPITFGQGTKVEIKRTVAAPSVFIF
PPSDEQLKSGTASVVCLLNNFYPREAKVQWKVDNALQS
GNSQESVTEQDSKDSTYSLSSTLTLSKADYEKHKVYACE
VTHQGLSSPVTKSFNRGEA

(numbering: 24, 34; 50, 56; 89, 97; 211, 215)

B

QVQLVESGGGLVQPGGSLRLSCAASGFTFTNYYMHWV
RQAPGKGLEWVSTIAYDGSSTYYADSVKGRFTISRDNS
KNTLYLQMNSLRAEDTAVYYCARWFTDDIWGQGTLVTV
SSASTKGPSVFPLAPSSKSTSGGTAALGCLVKDYFPEP
VTVSWNSGALTSGVHTFPAVLQSSGLYSLSSVVTVPSSS
LGTQTYICNVNHKPSNTKVDKKVEPKSEFLVPRGSGAP
EQKLISEEDLNDAPHHHHHH

(numbering: 26, 35; 50, 65; 95, 102; 217, 224)

C

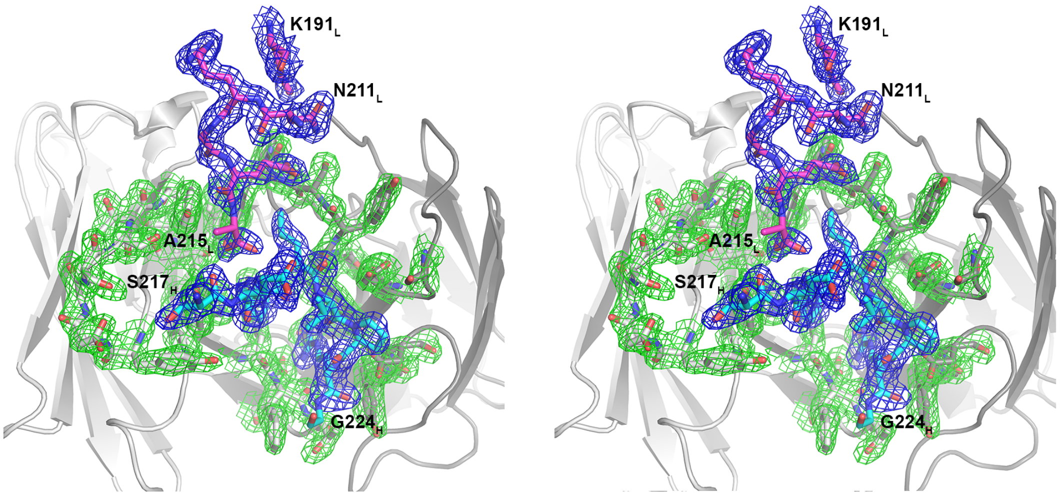

D

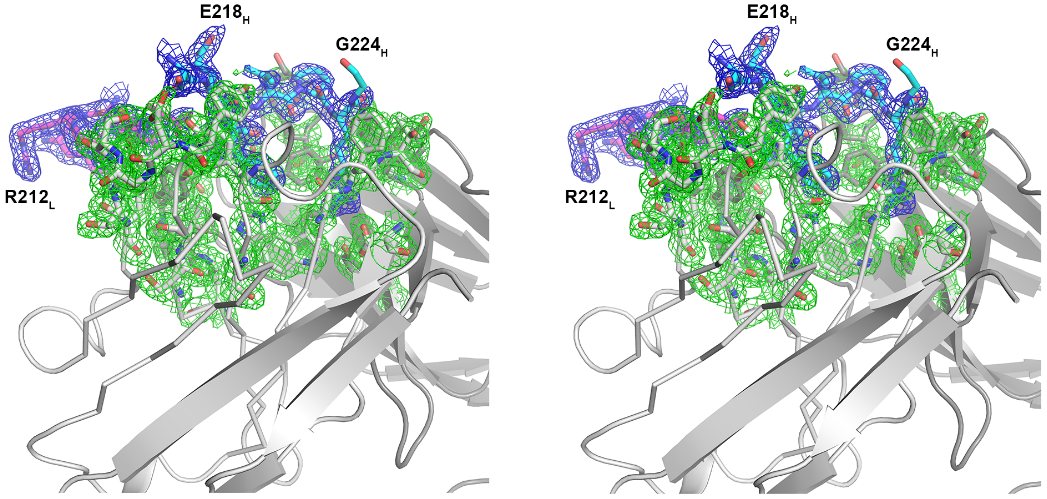

Supplementary figure 3: Amino acid sequences of the light (A) and the heavy (B) chain of the sclerostin-neutralizing Fab AbD09097. Complementary determining regions (CDRs) are indicated with the numbering following the convention according to Kabat. Red: CDR1, green: CDR2, blue: CDR3. The regions of the chain filling the antigen-binding crevice via crystal-lattice contacts are colored in cyan (heavy chain) and magenta (light chain). The amino acid sequence at the C-terminus of the Fab heavy chain representing the proteolytic site for thrombin is underlined, the succeeding sequences for the myc and hexahistidine peptide tags are in bold letters and boxed, respectively. (C) Detail showing the crystal lattice contacts between two symmetry-related AbD09097 molecules (stereo view) highlighting the quality of the weighted $2F_{obs}$-F_{calc} electron density map (showing the view top down the antigen-binding cleft). The two C-terminal sequences located within the antigen-binding cleft are colored in cyan and magenta (derived from the heavy and the light chain of a symmetry-related

AbD09097 molecule in the crystal lattice). The electron density map covering these two peptide regions is colored in blue, the electron density around the residual AbD09097 molecule is shown in green (carved to cover only the atoms shown in full detail). (D) As in (C) but rotated counter clockwise around the x-axis and the y-axis by 90°.

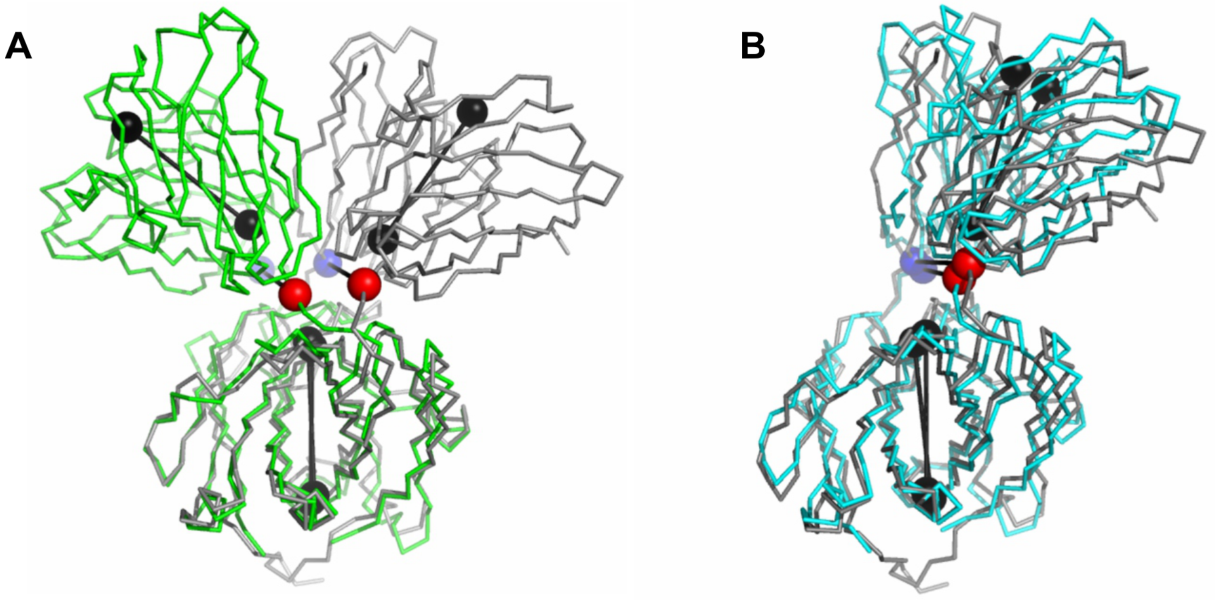

Supplementary figure 4: Structure alignment of AbD09097 (grey) with the BMPR-IA neutralizing Fab AbD1556 (PDB entry 2K3G, green) (A) and a Fab directed against K63-linked di-ubiquitin (PDB entry 3DVG, cyan) (B) to highlight the differences in the elbow angle between the constant and variable regions of the heavy and light chain. The dumbbells illustrate the elbow angles with the black dumbbells passing through the centers of mass of the variable and constant domains and the blue and red ones indicating the residues used to split the variable and constant domains (blue: light chain, red: heavy chain, figure created using Pymol script elbow_angle.py).

chain	sequence[a]	residue numbers[b]	CDR	Classification (North-AHO)	Classification (Chothia)
H	*ASS*|GFTFTNYYMH	26 - 35	H1	H1-13-1	class 1
H	TIAYDGSSTY**YADSVKG**	50 - 65	H2	H2-10-2	class 3-like
H	*AR*|WFTDDI	95 - 102	H3	H3-8-1	-
L	RASQSISSNELA	24 - 34	L1	L1-12-1	-
L	*Y*|DTSNRAT	50 - 56	L2	L2-8-1	class 1
L	QQYYSYPIT	89 - 97	L3	L3-9-cis7-1	class 1

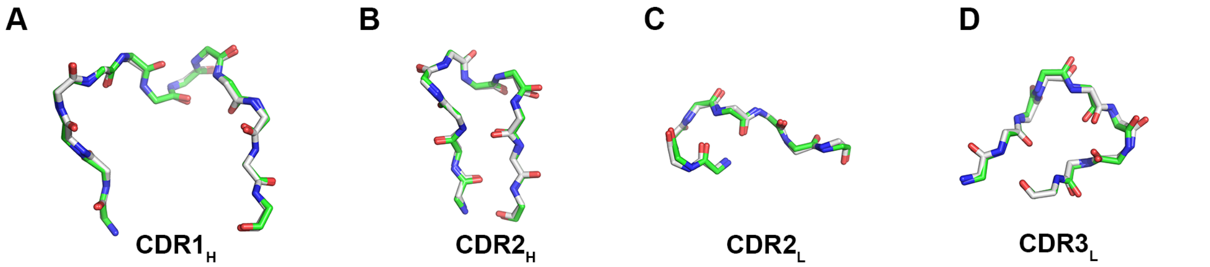

Supplementary figure 5: The table summarizes the classification of the CDR loops according to the scheme implemented by North and Honegger & Pluckthun (North-AHO, http://dunbrack2.fccc.edu) and by Chothia and co-workers (Chothia, http://www.bioinf.org.uk). [a] Loop boundaries vary depending on classification scheme. Sequences in italic were classified as CDR loop by North-AHO, but not when using the classification according to Chothia. Sequences in bold are considered part of a CDR loop by applying the classification of Chothia, but are not classified as CDR using North-AHO. [b] Numbering according to Kabat convention (see http://www.bioinf.org.uk). Panel of structural alignments of CDR H1 (A), CDR H2 (B), CDR L2 (C), and CDR L3 (D) with CDR loops of reference antibody structures as defined with the respective CDR loop classification according to Chothia and co-workers. Only backbone atoms are shown, residues of AbD09097 are shown with C-atoms colored in green, whereas residues of reference antibodies (PDB entries, 2FBJ (A), 1IGC (B), 1LMK (C), and 1TET (D)) are shown with C-atoms colored in grey.

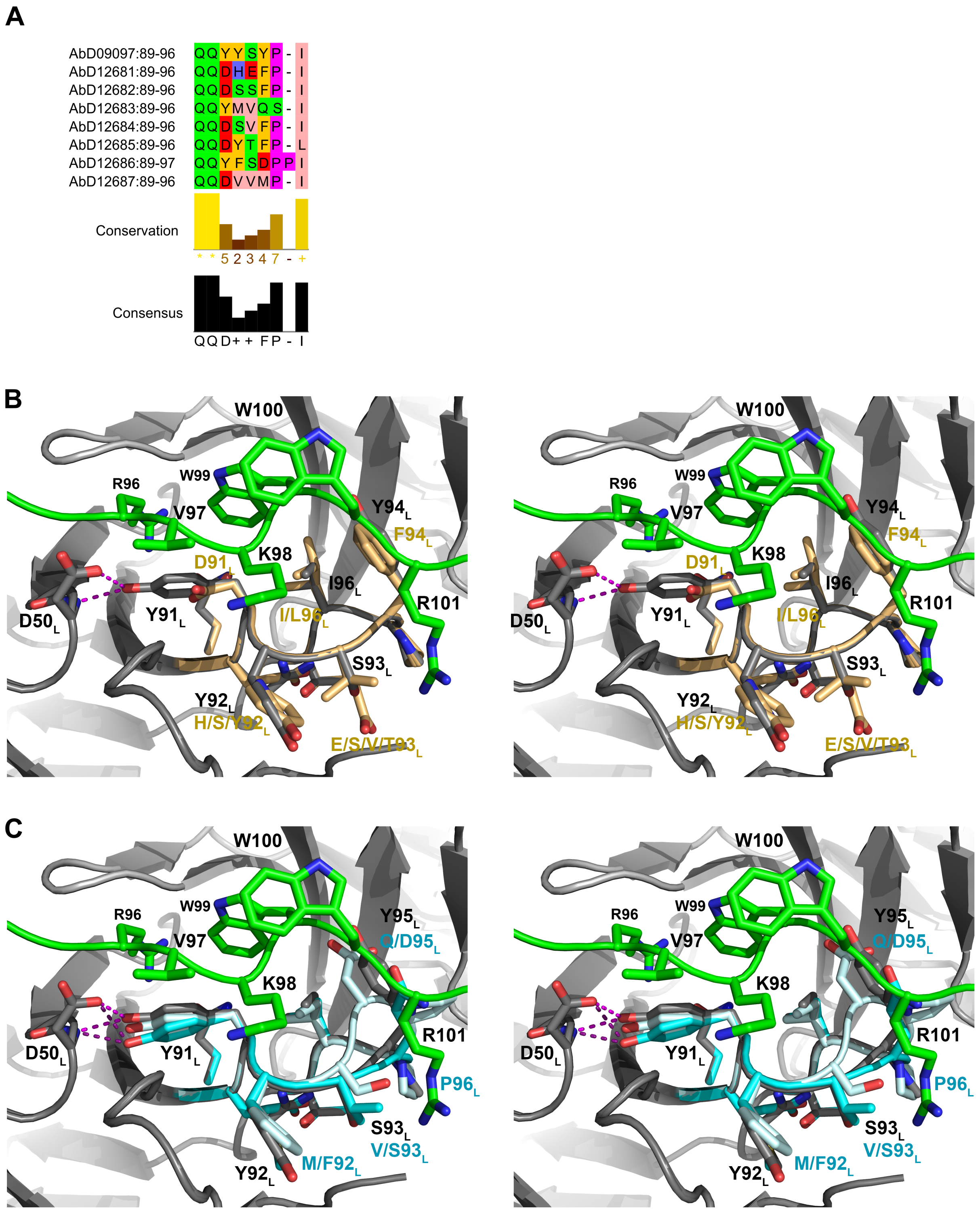

Supplementary figure 6: (A) Sequences of the light chain CDR3 of the ancestor AbD09097 and the affinity-maturated Fabs (AbD126..). The sequence alignment is visualized using the software Jalview and employing Zappo coloring scheme (http://www.jalview.org). The degree of conservation and the respective consensus sequence is shown. (B) Details of the interaction of the sclerostin loop 2 with the CDR3 of the light chain (CDR3$_L$). An overlay of the model of human sclerostin (residues Asn92 to Ser103) bound to AbD09097 (see figure 8) and the affinity-maturated Fabs AbD126.. is presented. The C-atoms of the loop peptide are colored in green, the amino acids in CDR3$_L$ of the ancestor AbD09097 are shown in dark grey. The differing amino acids of the affinity-maturated Fabs

AbD12681, 12682, 12684, and 12685, which are non-discriminatory between murine and human sclerostin, are indicated in light orange and marked accordingly. (C) As in (B) but for the AbD09097 derivatives AbD12683 and 12686 (C-atoms are colored in cyan and light blue, respectively), which bind highly specific to murine sclerostin with enhanced affinity.
